# Computed Tomography 3D Super-Resolution with Generative Adversarial Neural Networks: Implications on Unsaturated and Two-Phase Fluid Flow

**DOI:** 10.3390/ma13061397

**Published:** 2020-03-19

**Authors:** Nick Janssens, Marijke Huysmans, Rudy Swennen

**Affiliations:** 1 Department of Earth- and Environmental Sciences, Katholieke Universiteit Leuven, Celestijnenlaan 200E, 3001 Leuven, Belgium; mhuysman@vub.ac.be (M.H.); rudy.swennen@kuleuven.be (R.S.); 2 Hydrology and Hydraulic Engineering, Vrije Universiteit Brussel, Pleinlaan 2, 1050 Brussel, Belgium

**Keywords:** super-resolution, X-ray computed tomography, relative permeability, unsaturated flow, lattice Boltzmann method

## Abstract

Fluid flow characteristics are important to assess reservoir performance. Unfortunately, laboratory techniques are inadequate to know these characteristics, which is why numerical methods were developed. Such methods often use computed tomography (CT) scans as input but this technique is plagued by a resolution versus sample size trade-off. Therefore, a super-resolution method using generative adversarial neural networks (GANs) was used to artificially improve the resolution. Firstly, the influence of resolution on pore network properties and single-phase, unsaturated, and two-phase flow was analysed to verify that pores and pore throats become larger on average and surface area decreases with worsening resolution. These observations are reflected in increasingly overestimated single-phase permeability, less moisture uptake at lower capillary pressures, and high residual oil fraction after waterflooding. Therefore, the super-resolution GANs were developed which take low (12 µm) resolution input and increase the resolution to 4 µm, which is compared to the expected high-resolution output. These results better predicted pore network properties and fluid flow properties despite the overestimation of porosity. Relevant small pores and pore surfaces are better resolved thus providing better estimates of unsaturated and two-phase flow which can be heavily influenced by flow along pore boundaries and through smaller pores. This study presents the second case in which GANs were applied to a super-resolution problem on geological materials, but it is the first one to apply it directly on raw CT images and to determine the actual impact of a super-resolution method on fluid predictions.

## 1. Introduction

Fluid storage and flow properties are important to assess how water, oil, and gas are stored in rocks and how these fluids might migrate. In this way, it is possible to estimate for example how much CO_2_ could be captured in pores in the deep subsurface, which is crucial information if we want to reduce anthropogenic greenhouse gases by carbon capture and storage (CCS) [1,2,3,4,5,6]. Furthermore, it is also possible to study how waterflooding could enhance oil recovery from a reservoir [7,8,9]. However, fluid storage and migration is not only relevant for reservoir applications, but also for construction materials. Such materials may contain water that can be stored or removed during capillary uptake and drying, respectively. This water could enhance weathering and corrosion, form a medium for mould growth, or could expand upon freezing, thus breaking the rock [10,11]. Each of these preceding examples requires detailed knowledge on fluid storage and flow properties which could be estimated through experiments, either carried out in specialized laboratories or deduced from digital rocks. Laboratory measurements, however, require expensive and complex set-ups, take a long time to complete, are often destructive, do not always entirely capture the studied properties and on top of that, experiments often fail to produce reproducible results between laboratories [9,12,13,14,15,16,17,18,19]. Therefore, numerical solutions have been developed allowing to compute fluid storage and flow properties on digital rocks [7,20,21,22,23]. Such digital rocks are acquired through direct imaging or numerical simulations of rocks. 

Computed tomography (CT) is a technique to rapidly capture the three-dimensional structure of materials [22,24,25]. However, like any imaging technique, CT is plagued by a sample size versus resolution trade-off, which is adversely affecting fluid flow simulations. Both a high resolution and sufficiently large volume are required for accurate fluid flow estimations at the pore-scale [9,26,27,28]. Resolution impacts how well pore network topology and pore surfaces are resolved. At lower resolution, i.e., lower resolving power, pore sizes are often overestimated and specific surface area decreases. Additionally, fine pore connections like micropores, i.e., pores with sizes close or below resolution, might not be visualized while in reality they could be a determining factor for fluid flow. A combination of these factors leads to worsening fluid flow predictions with lowering resolution, even though total porosity tends to remain unchanged, especially for sandstones [20,26,27,29,30,31,32,33,34,35,36]. Unfortunately, it is impossible to only consider the highest possible resolution, because inevitably, a smaller volume would be imaged [24]. This smaller sample volume would risk zooming in on local heterogeneities, thus not studying a representative elementary volume, i.e., the minimal required volume to capture local heterogeneities that produces a homogeneous value for the studied property, like porosity or permeability [28,34,37,38,39]. This constant battle between using the highest possible resolution and the largest possible image size justifies the development of multiscale and super-resolution models. With respect to the latter, in this paper, an artificial resolution improvement method is presented.

Multiscale and super-resolution methods recently became more popular. Whereas some scientific questions concerning pore or grain size distributions can be answered by studying a single sample at multiple scales [15,16,40,41,42,43], others require the integration of different scales for fluid flow simulation. Several methods have been developed to integrate multiple scales, such as, multiscale pore networks in which fine pores are added explicitly to the pore network [44,45,46,47,48]. Instead of modelling fine pores explicitly, upscaled micropore properties could be added between macroscale pores as a continuum [49,50,51] or as local features, connecting pores parallel [52] or in series to macropore pore throats [23,53]. Apart from these pore network based approaches, which implicitly demand simplification of the pore structure via pore network extraction [22,54,55,56], multiscale images are another tactic to tackle the resolution versus sample size trade-off. Firstly, such multiscale models can be obtained through the superimposition of low and high resolution 2D images or 3D volumes. These 2D images are then obtained by extrapolating high resolution structures to larger images through two-point correlation functions and simulated annealing [57,58,59,60,61]. 3D volumes can be obtained from 2D images through process-reconstruction [62], two-point statistical relations [63,64], simulated annealing [59,65], phase recovery [66,67], and multiple point geostatistics [68,69,70,71,72]. These 3D models could then be superimposed to obtain a multiscale pore volume [48,73,74,75]. Secondly, multiscale images could be obtained by stochastically combining several scales and in contrast to image superposition, these models aim to jointly improve pore surfaces and fine-scale connectivity. Such multiscale images typically use expanding image grids to improve resolution through simulated annealing (SA) [61,76,77,78] or multiple-point geostatistics (MPS) [72,76]. Thirdly, super-resolution methods aim to introduce high resolution patterns into low resolution images by mapping a translation of low resolution features to high resolution equivalents. These methods could use self-similarity of images to improve details [79,80,81] or construct dictionaries linking low and high resolution image patch pairs through neighbour embedding [75,82] or sparse representation [83] by finding the closest matching high resolution image patch (e.g., 7 × 7 pixels) to a low resolution patch. These methods work in 2D using high resolution scanning electron microscopy (SEM) images and low resolution CT slices [75,82] and 3D, for which two CT scans at different resolutions were used [83]. Additionally, the translation from low to high resolution features could be addressed through neural networks [84,85,86,87]. Unfortunately, most of the preceding methods, especially those based on SA and MPS are rather slow in their performance and/or assume homogeneity of the simulated volume. These methods would be too computationally expensive to generate 3D simulations. This is problematic because porosity and fluid flow are 3D properties, thus they should be simulated in 3D. 

This research builds on the efforts of Wang et al. [82,83,85] and Shams et al. [88] to super-resolve, or improve the resolution, of lower resolution scans and it will more specifically be applying 3D generative adversarial neural networks. In order to have the most solid neural network, two CT scans taken at different resolution of exactly the same volume are used for training, because artificial downsampling of a high-resolution image to get a low resolution image is not performed without the risk of oversimplifying the problem [32,89]. The effect of oversimplification is further elaborated on in this research by studying the effect of mechanically versus artificially reduced resolution on the pore network and related fluid flow. Furthermore, even though the true strength of super-resolution methods should lie in the improved capacity to predict geophysical property predictions, like fluid flow, none of the super-resolution studies have yet addressed this and have instead limited model verification to peak signal-to-noise-ratios and structural descriptors (see, for example, [85]). Therefore, this research verifies whether fluid flow predictions for single-phase, unsaturated, and two-phase flow improved and studies the pore network characteristics to elucidate what improved. 

Convolutional neural networks were developed to mimic human sight and were initially mainly used for speech and image recognition [90,91,92]. Later on, these networks started getting used for super-resolution (SR) imaging [93] and some of the first applications focused on photographs (see, e.g., [93,94,95,96]) and movies [97]. They were rapidly used for the resolution enhancement of, for example, satellite images [98,99] and medical images [100], like magnetic resonance imaging [101,102] and CT [103,104,105,106,107]. The success of a lot of these SR applications is explained by the emergence of generative adversarial networks (GANs) [108] which are commonly known for their strength at generating realistic “fake” images [109]. These GANs have been used for geomaterial porosity simulation [110,111,112], but even though they have proven their capacity as super-resolution methods, they have not yet been applied to super-resolution of geological materials. In fact, neural networks have barely been used to solve this problem, except by Wang et al. [84,85] and Shams et al. [88]. Therefore, this research will apply 3D GANs to generate super-resolution models and it will do so by building on recent advances in super-resolution imaging. 

The goal of this research is to present a proof-of-concept study for super-resolution simulations, using generative adversarial neural networks (GAN) and to present how the developed approach influences fluid flow predictions. Next to these models, an explorative study was added to understand the impact of resolution on fluid flow properties of the studied continental carbonate. This resolution impact study was augmented by comparing samples that are scanned at different resolutions (mechanical resolution reduction) or samples that are artificially generated from a high-resolution volume (4 µm) which was artificially downsampled. Afterwards, suggestions are formulated that can help stabilize GAN training and future research directions are formulated to pave the path to deploy the developed super-resolution method for routine digital rock analyses.

## 2. Materials and Methods

### 2.1. Study Material

The studied material are continental carbonates, which have long been used as building material [113] and have recently been studied extensively as reservoir analogues [114,115,116] as a response to the discovery of pre-salt oil reservoirs consisting of similarly complex carbonates offshore in Brazil [117,118,119]. These carbonates provide unique challenges with respect to their porosity because an interplay of biological, chemical, and physical processes during sedimentation and diagenesis gives rise to an extraordinarily complex multiscale pore network, resulting in cm-scale pores that are connected to each other through meso- and microporous pathways [120,121]. One core plug of these continental carbonates has been used in this study. This sample was selected to have pores well below the sample size, although heterogeneity in pore structures was not avoided. This is further shown in Section 2.4. The studied sample has a diameter of 8 mm and is analysed at four different resolutions, i.e., 4, 8, 12, and 16µm. 

### 2.2. Computed Tomography

Computed tomography (CT) is a non-destructive technique that uses X-rays to obtain three-dimensional material reconstructions [24,25]. Three-dimensional volumes are represented by stacked 2D images in which grayscale variation is dictated by the Beer–Lambert law and is directly related to the attenuation coefficient of materials. These grayscales are then segmented into relevant material phases, which are, for this study, porosity and matrix (calcite) through hysteresis segmentation [122,123], in which two thresholds are selected and region growing corrects for misclassified voxels. These thresholds are manually selected by an experienced operator searching for the optimal balance between over- and underestimating connectivity. The major drawback of CT scanning is the resolution versus sample size trade-off, which is the major point of focus in this study. Three-dimensional visualization of these CT scans is obtained in Avizo Fire 9.0 software (ThermoFisher Scientific, Waltham, MA, USA).

### 2.3. Neural Networks

#### 2.3.1. Convolutional Neural Network–Network Architecture

**Figure 1:**
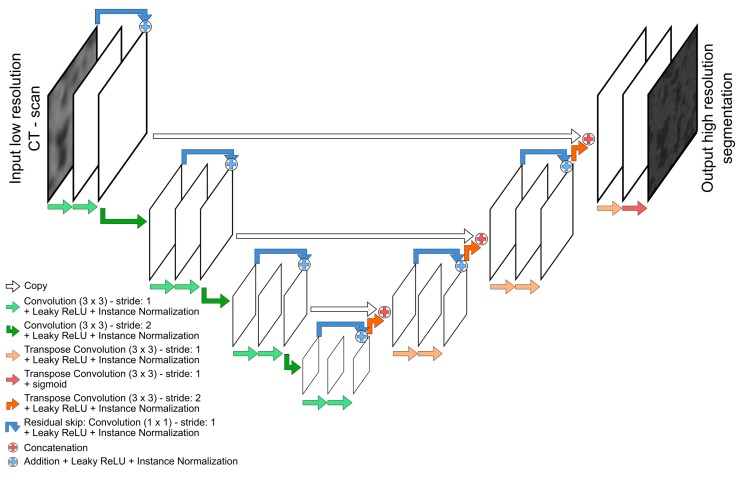
Fully convolutional neural network that was used for super-resolution Computed Tomography (CT) images. This sketch shows the followed path in only two dimensions, but in reality a three-dimensional approach was applied. The architecture is based on a U-Net [124], although short-range residual skips [127,129] have been added and span each convolutional block of three subsequent convolutions with as goal to speed up training.

Convolutional neural networks combine convolutional, pooling, activation, and normalization layers to mimic human sight and achieve tasks like image recognition and segmentation [90,91,92]. In this research, a fully convolutional network, and more specifically a U-Net is used [124]. U-Net refers to a type of encoder-decoder network in which a symmetrical network is used whereby one side downsamples the input through (strided) convolutions or pooling and the other side upsamples the images through (strided) deconvolutions or upsampling. Such U-Nets were originally developed for segmentation tasks in which the downsampling part of the network allows to consider a larger field-of-view with smaller convolutional kernels, thus including more contextual information. Without downsampling, only local information, limited to the kernel size would be considered. The U-Net used in this research is shown in Figure 1. The downsampling section consists of four convolutional blocks in which each block consists of two convolutional layers, each followed by leaky Rectified Linear Units (ReLU) activation layer [125] and instance normalization [126]. The leaky ReLU activation layer avoids complete deactivation of neurons which is typical for the more commonly used ReLU. In a ReLU activation layer, positive neuron values are passed, and negative values are set to zero (ReLU), or are decreased by a certain factor (Leaky ReLU). Instance normalization better handles image contrasts and depends less on batch size used to train the network, in contrast to more commonly used batch normalization. In between each convolutional block, a strided convolution with stride of 2 downsamples the volumes. Strided convolution allows additional trainable parameters for downsampling, whereas maxpooling, as used in original U-Nets [124] potentially removes important information. The upsampling section of the U-Net uses blocks with deconvolution and strided deconvolution layers to upsample the volume. The downstream and upstream part of the U-Net are connected by long-range skip connections, which concatenate feature maps of convolutional blocks to each deconvolutional block at the same level. This procedure recovers spatial information that could have been lost during downsampling [127]. In addition to these connections, each (de-)convolutional block contains a short-range skip connection, which enhances training convergence and mitigates the gradient vanishing problem [127,128,129]. The number of filters in each (de-)convolutional layer starts at 32 in the upper level and doubles each downward level, reaching a maximum of 256 filters in the bottom level. The final activation layer has sigmoid activation.

#### 2.3.2. Conditional Generative Adversarial Networks (GAN)—Super-Resolution Images

Generative adversarial networks consist of two convolutional neural networks of which one is the generator and the other is the discriminator [108,130]. The former network tries to generate realistic images, whereas the latter tries to differentiate these generated images from input training data. Both neural networks train to do their respective tasks better, but while the generator is getting better, it becomes more difficult for the discriminator the differentiate real and fake images. At the same time, a more effective discriminator increases the pressure on the generator to create more realistic images. Both networks are said to be playing a minimax game until a Nash-equilibrium has been reached in which both are sufficiently good at their tasks [108]. In reality, this usually means that the discriminator can correctly classify 50% of the images it receives [108,130,131]. The classification error is passed on to the generator as a measure of how bad it has functioned. 

**Figure 2:**
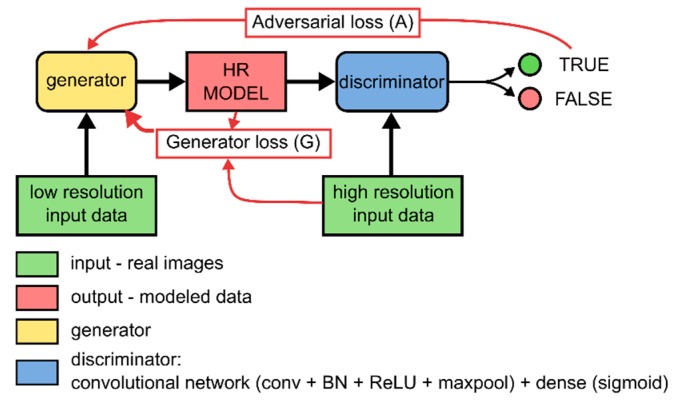
Conditional generative adversarial network (C-GAN) which was used for the super-resolution task in this research. A C-GAN consists of two neural networks, the generator and discriminator. The generator corresponds to the fully convolutional Residual U-Net shown in Figure 1 and the discriminator is a convolutional network combined with a dense network tasked to classify real and “fake” images. The goal of this C-GAN is to train the generator to simulate high-resolution (HR) equivalents of low-resolution (LR) input volumes by training using a generator and an adversarial loss.

Typically, GANs create images from a random latent vector but in a conditional GAN an input image is given to the generator, which is then modified to create the desired image [132]. In this scenario, an additional loss function, apart from the discriminator loss is passed onto the generator to train. This additional loss function directly compares the generated image to the expected image (Figure 2). 

In this research, the generator corresponds to the same U-Net as explained before. The discriminator is a convolutional network, with four strided convolutions (stride = 2) each followed by leaky ReLU activation and instance normalization. The first convolution starts with 64 filters and this is doubled for each subsequent convolution. These four convolutions are followed by a fifth convolution (stride = 1) with one filter, which is then flattened and followed by a dense network with one hidden layer (1024 neurons) followed by leaky ReLU activation, 50% dropout and a single output neuron with sigmoidal activation. This network decides whether the input images are real or generated by the generator. 

GANs often show unstable training behavior. Therefore, the suggestions of Radford et al. [130] were followed by opting for strided convolutions instead of pooling and normalization and leaky ReLU activation layers after each convolution. Apart from this, network complexity was improved stepwise, adding a new level of network depth after each increment. Furthermore, residual skips over each convolutional block helped increasing stability. In order to reduce the effect of having insufficient pattern variability, the batches that were used to train the discriminator were randomly shuffled.

#### 2.3.3. Training

The generator of the GAN is trained with generator loss, which is a mean absolute error (mae) loss and with adversarial loss which is obtained by feeding the discriminator “fake” images, but computing the mean squared error (mse) loss by labelling the images as real. If these images are labelled as “fake” by the discriminator, the generated did not fool the discriminator. Therefore, the loss will be higher, thus motivating the generator to improve. The generator and adversarial loss are fed to the generator in a 100:1 ratio. The input parameters are summarized in Table 1 and are the result of testing common input parameters found in the literature on medical CT applications [133] through a grid search approach on 2D test sets. The number of epochs was selected based on a trade-off of artefacts versus simulation time. Training is performed on a user-grade NVIDIA GeForce GTX 1080 graphical processing unit (GPU) with 8 GB of graphical memory. Furthermore, the networks are developed in Keras with a TensorFlow 1.14 backend.

### 2.4. Dataset Preparation

Four scans at resolutions of 4, 8, 12, and 16 µm, respectively, have been taken of a 8-mm core plug. From each of these scans the same volume is extracted, with dimensions of 4.8 mm × 4.8 mm × 6.6 mm, which corresponds to 1200 × 1200 × 1750 voxels in the 4 µm scan. In order to maximize the match of these volumes and to ensure that exactly the same volumes are compared for each resolution, the volumes at lower resolutions are cut by comparing bounding box slices through structural similarity [134]. These four volumes are used to verify the influence of resolution on pore network and fluid flow properties. Additionally, the 4 µm resolution scan has been downsampled by factors 2, 3, and 4 in order to obtain volumes at 8, 12 and 16 µm voxel size, respectively. Artificial resolution reduction or downsampling was performed by averaging voxel values of segmented images by, e.g., a mean filter of 2 × 2 × 2 to get resolution reduction from 4 to 8 µm. This procedure is preceded by a spline filter. This process allows comparison between artificially reduced resolution (ARR) and mechanically reduced resolution (MRR) and helps to establish whether ARR could suffice to train neural networks for large-scale deployment.

**Figure 3:**
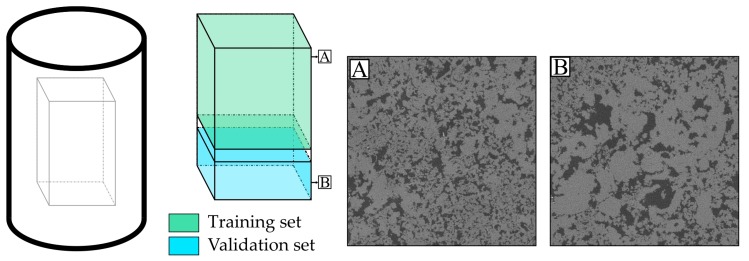
Dataset preparation. The same prism is extracted from each input volume, which are four scans at resolutions 4, 8, 12 and 16µm, respectively. Then, from the 4 and 12 µm scans, training and validation data was extracted for training of the super-resolution C-GAN. Training and validation data are non-overlapping and not adjacent to ensure sufficient variability between the pore network, as to avoid overfitting of the neural networks. **A** and **B** show that pore structures can significantly change within one sample. **A**. has smaller, regularly distributed pores, at the top of the sample and **B** has more pore size variation in the lower part of the sample.

Two of the scanned volumes are used to train and validate the neural network, namely the volumes scanned at 4 and 12 µm resolution at high (HR) and low resolution (LR), respectively. These volumes are then divided into training data, 1000 slices, and validation data, 600 slices (Figure 3). The remaining 150 slices in between these two volumes, because the original volume had 1750 slices, are aimed to reduce similarity between training and validation data. Despite this potential similarity, it was preferred to take training and validation data from the same sample because grayscale contrast differences could occur for different samples, which are avoided for this proof-of-concept study. Additionally, continental carbonates are known for their complexity, thus making the lower and upper part of one sample sufficiently different (Figure 3). For example, in Figure 3, the upper part of the sample dominantly contains finer, regularly distributed pores, whereas the lower part has more irregularly distributed pores. The LR training and validation volumes are upsampled by approximately a factor of 3 to match the grid size of low and high resolution volumes, although the correct factor is determined from the volume matching procedure. This brings LR and HR data to the same voxel size. Finally, the validation and training datasets are subdivided into subvolumes of 64³ voxels and each of those volumes overlap by 25%. Such small volumes are required to allow training of the neural network on a single-GPU device, and the overlap is used to enhance the amount of samples in the training set. Validation is performed on larger volumes of 256 × 256 × 64, again with an overlap of 25%. Larger volumes and overlap are chosen to reduce stitching artefacts. Samples were not preprocessed elaborately as the future intended goal of these neural networks are routine use, independent of preprocessing. However, grayscale values were normalized between 0 and 1 so as to avoid exploding gradients in neural networks. 

A validation dataset is provided to test whether the models could work on previously unseen data. This dataset also consists of a HR and LR volume. During validation, only the LR volume is fed to the GAN to create a super-resolved model (SRM). These SRM and the related fluid flow properties can be compared to those of the available HR volume.

### 2.5. Fluid Flow Properties

Fluid flow properties have been deduced from the subsamples of the studied CT scans. These subsamples have dimensions of 1000³ voxels to study the influence of resolution and 500³ voxels to study the efficiency of super-resolution GANs, compared to the original LR and expected HR volumes. For the resolution study, two volumes (V1_r_ and V2_r_) were extracted at each resolution with an overlap of 40%. The volumes are taken at the exact same location at each resolution. To study the effect that super-resolution GANs can have after transforming LR data, four volumes (V1_SRM_, V2 _SRM_, V3 _SRM_ and V4 _SRM_) were extracted from LR, HR and super-resolved models (SRM), with maximal overlap of 20%. Here, these four volumes are taken at the exact same spot. The volume size difference for the two studies is caused by the dimensions of modelled volumes, which are 1156 × 1156 × 576 voxels because validation was performed on the validation set which was not involved in training of the neural network, as to avoid overfitting (Figure 3). For the resolution study, the entire 1200 × 1200 by 1750 voxels cube was used. The different volumes, i.e., V1_r_ and V2_r_ or V1_SRM_, V2_SRM_, V3_SRM_, and V4_SRM_ are not compared to each other as this would imply that a representative elementary volume (REV) was reached and computation of the REV was not part of this study.

#### 2.5.1. Lattice Boltzmann Simulation—Single-Phase Permeability

Lattice Boltzmann simulations (LB) are used to solve the discretized Boltzmann equation to simulate fluid flow on a regular lattice [135,136,137]. The D3Q19 lattice was selected to balance accuracy and computability [138,139]. LB simulations were performed in the Palabos open-source package [140] with an applied pressure difference of 0.0005 Pa [72]. Results are visualized with Paraview [141,142]. Additionally, porosity and specific surface of models are computed by counting porosity voxels on segmented CT volumes to obtain the total pore volume and through marching cubes algorithm for surface area. This surface area is then translated to specific surface area by dividing the surface area by pore volume. 

#### 2.5.2. Pore Network Models (PNM)

Fluid flow simulations are most easily computed on pore network models. These models present a simplified representation of the pore structure, in which the pore network is simplified into pores and pore throats, represented as balls and sticks. The network extraction algorithm of Raeini et al. [55] was used to generate input for fluid flow simulations. On these extracted pore network models (PNM), single-phase absolute permeability, two-phase relative permeabilities and unsaturated flow properties were determined.

#### 2.5.3. Unsaturated Flow: Moisture

Unsaturated moisture flow is often used for building materials and looks at moisture retention and flow in materials. These properties are of prime importance in ensuring safe building materials by avoiding mould growth and corrosion. Islahuddin and Janssen [10,19,143] developed unsaturated fluid flow simulations, which are directly applied in this research, using modified OpenPNM code [144] to simulate moisture retention and permeability in relation to capillary pressure, *p_c_* through the Young-Laplace equation, modified by Bakke and Øren [62]:

(1) $$p_{ct}=\frac{-\left(1+2\sqrt{\pi G}\right)\;\sigma \cos\theta }{r}$$

where *θ* is the contact angle, *σ* is surface tension, and *G* is the shape factor.

A good explanation of all used equations and physics behind the computed properties are found in Islahuddin and Janssen [19]. In the present research, air entrapment, corner and adsorbed flow, and Knudsen diffusion are included, even though Knudsen diffusion should not make a large difference because of the relatively large pores encountered in this study.

#### 2.5.4. Two-Phase Flow: Oil and Water

Two-phase flow simulations are often used to model displacement of water by oil or oil by water during primary drainage or waterflooding, respectively. Usually, PNM are used for these simulations, as direct simulations require careful tracking of fluid interfaces making them computationally expensive [9]. Therefore, this research applies the code of Valvatne [145] and Valvatne and Blunt [7] to simulate two-phase fluid flow on PNM by considering primary oil flooding and subsequent water flooding. The rock is initially assumed to be water-wet, although after oil flooding, pore boundaries in contact with oil become oil wet, making the carbonates mixed-wet [7,9].

In these two-phase simulations, capillary pressure corresponds to the pressure difference between water and oil. Simulations will run until a certain fraction of oil-filled pores is attained or until all pores are filled with oil. During primary oil flooding, the only relevant flow mechanism is piston-like displacement [146] and pore entry capillary pressures (*p_ce_*) are given through the Young–Laplace equation, modified by Mason and Morrow [147] and Øren et al. [56]:

(2) $$p_{ce}=\frac{\left(1+2\sqrt{\pi G}\right)\;\sigma \;\cos\theta_{r}}{r}\;F_{d}\left(\theta_{r},\;G,\;\beta \right)$$

where *θ_r_* is the receding contact angle and *β* the corner half-angle. The main difference with the unsaturated threshold capillary pressure (Equation (1)) relates to the *F_d_* as dimensionless correction factor, accounting for liquid that remains in pore corners, further elaborated in Valvatne and Blunt [7]. The receding contact angle is in its turn depending on factors like surface rugosity, wettability, and mineralogy [148].

## 3. Results

The result section combines the pore network and fluid flow properties that were obtained to study the resolution influence and implementation of super-resolution models (SRM). Both are analysed using the same techniques, resulting in similar tables and figures, thus please take attention to the figure axes and table headers. When comparing low resolution input, super-resolved models and the expected high-resolution output, the abbreviations LR, SRM, and HR will be used, respectively. Furthermore, whenever results are visualized, the results of only one volume are used, but all properties show similar variability, as is demonstrated by supplying all quantitative values in table format for each volume. Furthermore, all results are plotted in the Supplementary Material for the reader to assess the similarity between all volumes. Whenever the term “resolution” is used, the voxel size is implied and not resolvability. 

### 3.1. Visual Results

**Figure 4:**
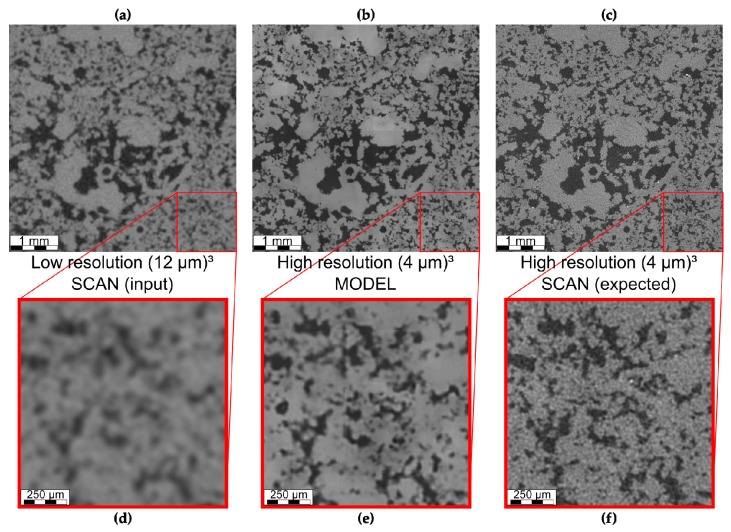
Visual results comparing the low resolution (LR) input (**a**,**d**) to the output (SRM) of the super-resolution GAN (**b**,**e**) and to the expected high resolution (HR) output (**c**,**f**). The blurriness of LR has disappeared in SRM and small pores are more clearly delineated, similar to HR. Furthermore, both LR and HR contain noise, which has disappeared in the SRM. However, SRM and HR do not have a one-to-one match.

**Figure 5:**
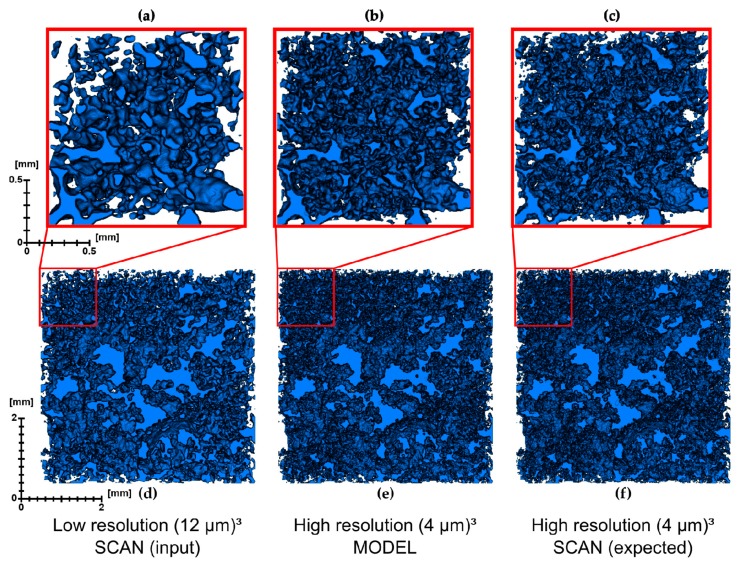
Three-dimensional volumes comparing LR input (**a**,**d**) to the super-resolved model (SRM—**b**,**e**) and the expected HR volume (**c**,**f**). The LR volume gives the impression of containing less pores and smoother pore edges. SRM appears to have more pores and more complex pore boundaries, compared to the LR input, although it does not yet fully equate to the expected HR. However, visually SRM is more closely resembling HR than LR.

Slices of LR input, expected (HR) result and SRM are compared in Figure 4. The SRM (Figure 4b,e) has reduced salt and pepper noise, compared to HR (Figure 4c,f). Furthermore, zones with grayscale values in between porosity and matrix are influenced by the partial volume effect, and are better resolved in SRM than in LR, as they as are in HR as well. However, the SRM pores do not necessarily match HR one-to-one. This is shown in detail in Figure 4d–f. Figure 5 shows a three-dimensional visualization of the LR input, expected HR result, and SRM after segmentation.

### 3.2. Pore Network Properties

**Figure 6:**
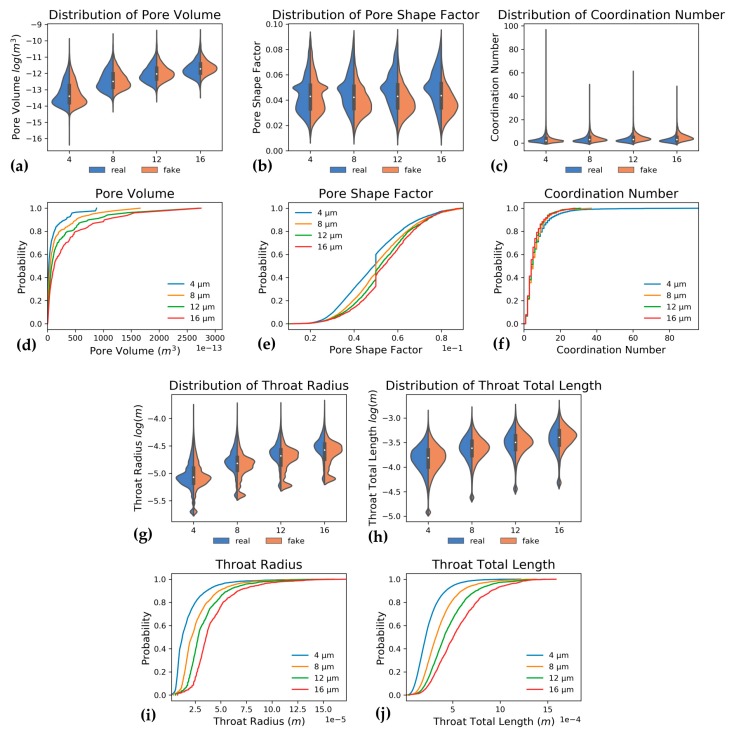
Study of resolution effect. Pore network properties showing the influence of resolution (in µm) in cumulative distribution plots, while violin plots also show how these properties vary for artificial (fake) versus mechanical (real) resolution reduction. In cumulative distribution plots, only the mechanically reduced resolution is shown. Data from first volume (V1). Pore volume (**a**,**d**) increases with decreasing volume, pore shape factor (**b**,**e**) increases for MRR (real) and decreases for ARR (fake), coordination number (**c**,**f**) is constant for MRR (real) and raises for ARR (fake). Pore throat radii (**g**,**i**) and lengths (**h**,**j**) decrease with improving resolution.

Pore network properties are determined on pore network models extracted from segmented images. The influence of resolution on these properties is shown in Figure 6 for V1 and in Table 2. This table distinguishes real mechanical resolution reduction (MRR), as obtained from scanning at multiple resolutions and artificial resolution reduction (ARR), which was obtained from downsampling the sample scanned at 4µm. Note that all data shown with respect to lengths and volumes are transformed through log_10_ transformation. The ratio of pore throats to pores remains constant at approximately 1.60 for MRR, while this ratio increases from 1.60 to 2.75 for ARR. Similarly, the coordination number increases from 3.13 to 5.38 for ARR while remaining constant around 3 for MRR (Figure 6c,f). The mean pore shape factor increases from 0.045 to 0.052 for MRR while decreasing to 0.039 for ARR (Figure 6b,e). The pore and throat shape factor distributions (Figure 6) show that pores and pore throats with lower shape factor are disappearing for real volumes (Figure 6b), while in “fake” volumes, this shape factor distribution becomes heavier towards lower pore values and remains rather constant for pore throats. All other length related properties, like pore and throat radii, volumes and lengths, increase on average, both for ARR and MRR, although violin plots (Figure 6a–c,g,h) show that artificial volumes contain relatively more smaller pores and pore throats than MRR volumes. Additionally, Figure 6 shows that the amount of pore throats with the smallest possible throat radius (Figure 6g), equal to 1 voxel or the resolution, is much higher for ARR than for MRR. 

**Figure 7:**
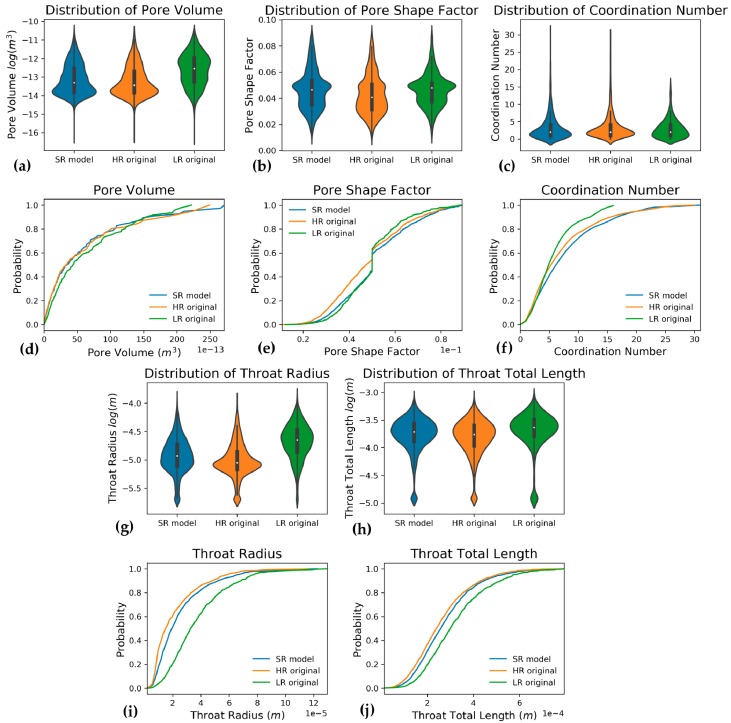
Study of effect of SR models. Pore network properties of Low Resolution model input, super-resolved (SR) model output and the expected high resolution output in cumulative distribution plots and violin plots. Only data from first volume (V1) is shown. Pore volume distribution (**a**,**d**) is closer to HR for SRM than LR and pore shape factor distribution (**b**,**e**) of SRM is in between distributions of LR and HR. Coordination number (**c**,**f**) is better approximating HR in SRM than in LR. Pore throat radii (**g**,**i**) and lengths (**h**,**j**) are also better reproduced by SRM than LR can show.

Pore network property distributions comparing LR, HR, and SRM, as well as the cumulative distributions of these properties are shown in Figure 7. Furthermore, average values of pore network properties are summarized in Table 3. Compared to LR input, SRM contains almost as many pores and pore throats as the expected HR output does. At least, the ratio of pore throats per node is better represented. Further, all other properties, like coordination number and pore volumes as well as throat radii and lengths of SRM more closely represent HR data than the LR data. In addition to these mean values, violin plots and cumulative distributions (Figure 7) show that pore volumes (Figure 7a,d), throat lengths (Figure 7h,j) and radii (Figure 7g,i) and coordination numbers (Figure 7c,f) of SRM are more similar to HR than they are to input LR. Conversely, the pore shape factor mean and distribution remain more similar to LR despite the different pore counts (Figure 7b,e). Additionally, throat radii are larger in SRM than they are for HR, although they present an improvement compared to the larger radii in LR input.

Additional pore network properties which are not explicitly shown in this sections, like pore radii, throat volumes and throat shape factors are added as supplementary material (Supplementary Figure S1 for resolution influence and S2 for model validation), as each of these properties provides similar results to its pore or pore throat counterpart. 

### 3.3. Porosity and Absolute Permeability

The influence of resolution on porosity, specific surface area (SSA) and absolute permeability estimated from pore network models, is shown in Table 4. With increasing resolution, porosity decreases slightly for MRR, from 0.26 to 0.23, while it remains constant for ARR. The specific pore surface area halves for MRR, while it only decreases up to 20% for ARR. Permeability is increasing with worsening resolution and this increase is more pronounced for MRR than for ARR. 

**Figure 8:**
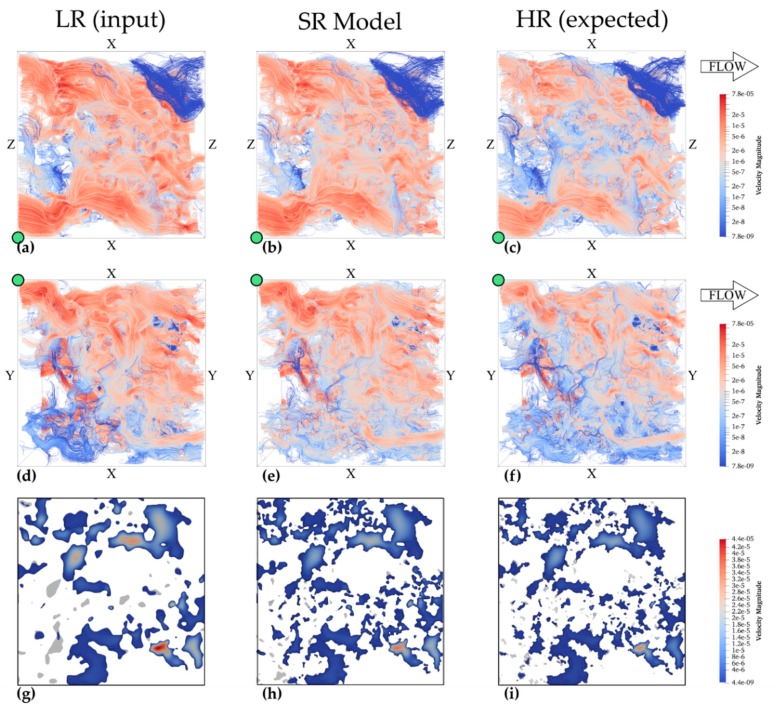
Visualization of lattice Boltzmann simulations in 3D along a side view (X-Z) and a bottom view (X–Y) in which the green dots indicate the same point in each volume that was added for orientation. Fluids flow from left to right and red indicates higher fluid flow velocities, while blue indicates slower fluid flow. In the top row (**a**–**c**), the HR sample (**c**) has two higher fluid velocity zones with a lower fluid velocity zone in the middle. This distinction is less obvious in LR sample (**a**), while the slightly bluer colour in the middle of SRM (**b**) suggests a certain improvement of the model. The middle row (**d**–**f**) show an unconnected zone in the lower left corner of the LR sample (**d**), which is again connected in the SRM image (**e**), as it should be, as shown in (**f**). The bottom row (**g**–**i**) shows a 2D slice perpendicular to this flow and is taken in the middle of the sample. In this slice, the grey colours indicate unconnected porosity. These slices show that the SRM (**h**) have more pores and are better connected than the LR volume (**g**). However, connectivity and fluid flow velocities are still exaggerated compared to HR (**i**). This is also seen from the 3D volumes where SRM volumes (**b**,**e**) have a redder colour than HR (**c**,**f**), although it is slightly more blue than LR volumes (**a**,**d**).

The effect of super-resolution modelling (SRM) on porosity, SSA and permeability is shown in Table 5. These results show a consistent porosity overestimation between 9% and 13% compared to the expected HR data. However, permeability is better estimated by SRM than it was for LR, despite a remaining overestimation between 39% and 91%. LBM-simulated permeability closely matches PNM-derived permeability and a visualization of these models is shown in Figure 8. In this figure, the bottom row shows a 2D cross-section perpendicular to the flow direction and shows pores that are not transporting any fluids (Figure 8g–i). A larger fraction of pores is disconnected in (LR—Figure 8g) with higher fluid velocity in the largest pores, in comparison to the other two volumes. However, SRM shows additional pore connections and slightly higher fluid velocities compared to HR. In general, HR shows a low velocity zone at the borders of flow paths (Figure 8c,f,i), thus at the borders of pores, whereas these low velocity zones are less represented in LR. The SRM fits in between these two volumes as lower velocity zones are better represented, although fluid flow velocities tend to be higher than in HR (Figure 8b,e,h). The SSA is underestimated by approximately 10% in SRM in comparison with HR, but this is a serious improvement to the 40% underestimation in LR.

### 3.4. Unsaturated Flow

**Figure 9:**
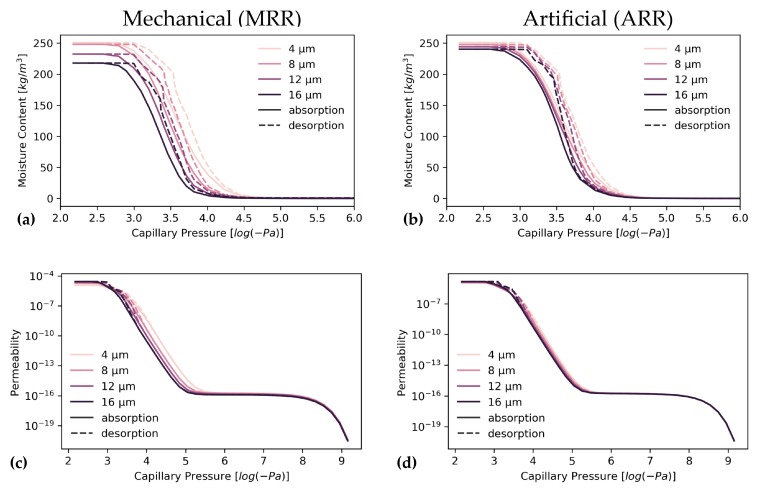
Resolution influence on unsaturated moisture absorption and desorption or drying (**a**,**b**) and moisture permeability (**c**,**d**) with changing capillary pressure. Absorption would increase capillary pressure because this pressure is linked to relative humidity thus following the graphs from right to left. Desorption or drying follows the opposite direction. The difference between the solid and dotted line shows ”hysteresis”. Only data from first volume (V1) is shown. Note that **a** and **b** have their x-axes cropped to represent a narrower capillary pressure range than moisture permeability (**c**,**d**), because no visible moisture variation occurs in the higher log(-Pa) range. In samples obtained through Mechanical Resolution Reduction (MRR), the spread on the onset of moisture increase (**a**) and permeability increase (**c**) is higher than for samples obtained from Artificial Resolution Reduction (ARR), as seen for moisture uptake (**b**) and permeability increase (**d**).

The influence of resolution on unsaturated flow is shown in Figure 9. This influence is most pronounced in the onset of moisture content increase at lower capillary pressures for the highest resolutions (Figure 9a,b). Maximal moisture is achieved at similar capillary pressures for all resolutions. The highest resolution sample has the highest saturated moisture content and this gradually decreases with worsening resolution. Furthermore, drying onsets more or less at the same capillary pressure, but shows the smoothest curve at the highest resolution. This highest resolution sample is also the last to be completely dry. Permeability follows a similar course to moisture increase as the onset of permeability increase occurs at lower capillary pressure for the highest resolution sample but levels out at the same capillary pressure (Figure 9c,d). However, despite lower saturated moisture content at the lowest resolutions, final permeability is higher at these low resolutions. All these observations are much less pronounced or even invisible for ARR (Figure 9 and Supplementary Figure S3).

**Figure 10:**
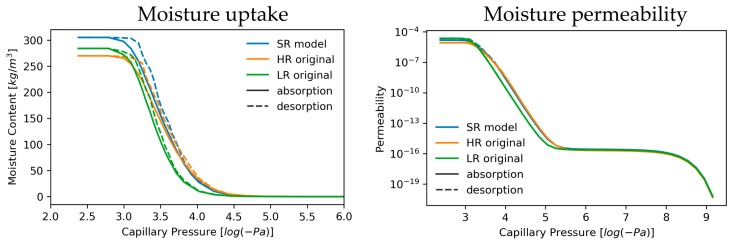
Impact of super-resolved models (SRM) on unsaturated moisture absorption and desorption on moisture permeability (right) and drying (left) with changing capillary pressure. Absorption would increase capillary pressure because this pressure is linked to relative humidity thus following the graphs from right to left. Desorption or drying follows the opposite direction. The difference between the solid and dotted line shows ”hysteresis”. Only data from first volume (V1) is shown and note that the left graph has its x-axes cropped to represent a narrower capillary pressure range than moisture permeability (right plot), because no visible moisture variation occurs in the higher log(-Pa) range.

Unsaturated flow properties for SRM validation are shown in Figure 10. During moisture uptake, moisture increases at lower capillary pressure, approximately −10^4.6^ Pa, for SRM and HR data than for LR, where moisture starts collecting at approximately −10^4.3^. This same observation is made during drying where drying starts sooner for LR than for HR and SRM reconstructions. However, saturated moisture content is highest for SRM and lowest for HR (Figure 10, Supplementary Figure S4, Table 6). Moisture permeability shows a similar pattern, in which permeability rises more rapidly for SRM and HR, while it is later for LR. In Table 6, quantitative values are shown to compare moisture permeabilities at maximal saturation and at the plateau between −10^8^ to −10^6^ Pa, which represents vapour permeability. This vapour permeability is overestimated by 27%–37% for SRM whereas it is better estimated by LR with a 8%–18% difference from HR estimates. In contrast, maximal permeability is better represented by SRM with overestimations of 71%–106%, while LR overestimated this permeability by 118%–270%.

### 3.5. Two-Phase Flow

**Figure 11:**
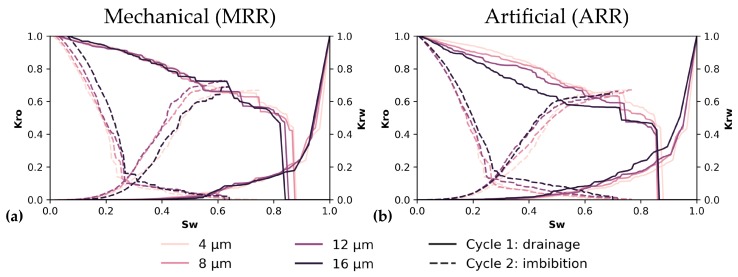
Resolution influence on relative permeability evolution of oil (K_ro_) and water (K_rw_) with varying water saturation (S_w_) during oil-flooding (drainage) and water-flooding (imbibition). K_ro_ decreases with increasing water saturation and K_rw_ increases. **a** shows the evolution of relative permeabilities for the same volume at four different resolutions obtained from Mechanical Resolution Reduction (MRR) and in **b** this is shown for Artificial Resolution Reduction (ARR).

**Figure 12:**
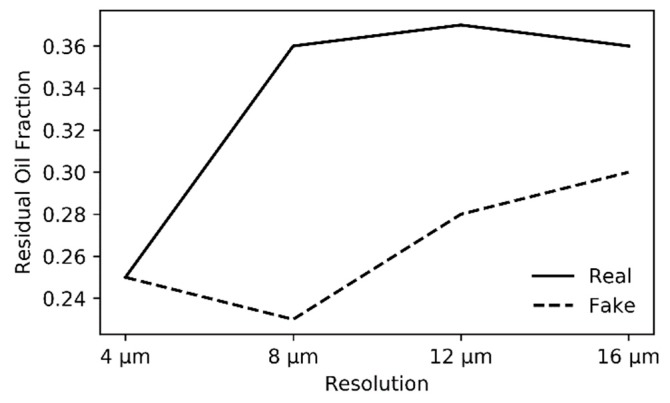
Resolution influence residual oil fraction after waterflooding, studied for mechanical (real) and artificial (fake) resolution reduction.

Two-phase fluid flow simulations have been performed in two cycles, i.e., oil-flooding and water-flooding. The effect of resolution on these simulations is shown in Figure 11. Most of these profiles look similar over all resolutions, both for MRR (Figure 11a) and ARR (Figure 11b). However, the major difference is visible during water-flooding (oil extraction), where residual oil is significantly different for the different resolutions and the effect of MRR is clearly more pronounced, as shown in Figure 12.

**Figure 13:**
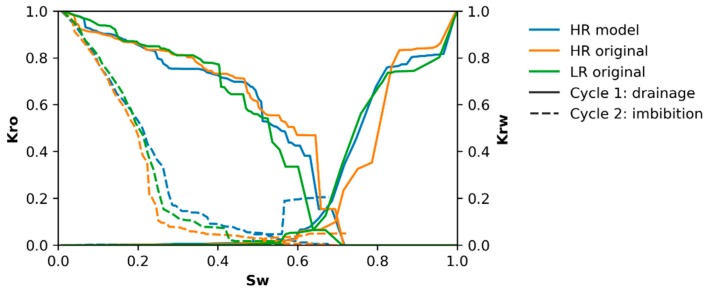
Impact of super-resolved models on relative permeability evolution of oil (K_ro_) and water (K_rw_) with varying water saturation (S_w_) during oil-flooding (drainage) and water-flooding (imbibition).

The effect of two-phase flow on SRM is shown in Figure 13 for V1_SRM_, and other volumes are shown in the supplementary materials section (Supplementary Figure S5). In this image, drainage (oil-flooding) and imbibition (water-flooding) largely have similar profiles for the three samples (LR, HR and SRM), although the final amount of residual oil varies between 25% and 36% for the high resolution (HR) volumes, between 41% and 46 % for low resolution (LR) volumes, and between 32% and 35% for super-resolved models (SRM), as shown in Table 7. This results in relative overestimations between 15% and 40% for SRM, with one sample underestimating residual oil fraction by only 4%. This is less than the 15%–82% overestimations for LR. Furthermore, huge variability is observed for final water permeability after waterflooding, with only two of the four volumes having estimates of both LR and SRM within the range of HR. In these two cases, the SRM better predicted this value than the LR volumes, and the latter significantly underestimated this final water permeability. In the fourth volume, K_rw_ appears severely overestimated in SRM, although here K_rw_, is almost non-existent for HR, LR, and SRM. Additional plots are shown in Supplementary Figure S5.

### 3.6. Training and Simulation Speed

The models trained approximately eight days on a user-grade NVIDIA GEFORCE GTX 1080 graphical processing unit (GPU) with 6800 training volumes and a batch size of four samples. After training, 1.73 volumes with dimensions 256 × 256 × 64 voxels could be simulated per second on this same GPU with a batch size of 1 sample. Processing these same volumes with solely a CPU rendered simulations much slower, taking 11 s for each 256 × 256 × 64 voxel volume.

## 4. Discussion

In this study, three-dimensional generative adversarial (neural) networks were used to achieve super-resolution CT scans. The three-dimensional results were analysed through pore network extraction and subsequent fluid flow simulations for single-phase, unsaturated and two-phase flow. Comparisons for fluid flow simulations are only possible by comparing to results from the highest resolution image because no experimental data is available. Therefore, it will be assumed that simulations at the highest resolution provide the most realistic and accurate results. This assumption is validated by observations made by Shah et al. [32] for two-phase flow and Arns et al. [20], Alyafei et al. [26,30], and Guan et al. [33] among others for single-phase flow. However, the influence of resolution on all studied parameters has been analysed at four different resolutions for the presently studied sample to put this super-resolution analysis in a framework and to understand how decaying resolution impacts each studied parameter.

### 4.1. Resolution Influence

The influence of reduced resolution has been studied for mechanical (MRR) and artificial (ARR) resolution reduction. The former is the result of either scanning larger samples or shifting the sample further from the X-ray source while keeping the source-detector distance constant. The latter, artificial resolution reduction, downsamples a sample scanned at a resolution of 4 µm through average voxel binning. ARR is interesting because it creates perfectly coupled volumes at several resolutions which is the ideal dataset for training a neural network, such as the super-resolution GAN used in this research. However, in order to deploy such SR-GANs to real situations, the training data should be representative of whatever input would be provided. Therefore, mechanical and artificial resolution decrease is compared to verify whether ARR sufficiently captures quality loss of actually scanned samples. Additionally, this study of the impact of resolution on fluid flow properties validates the possible need for super-resolution models. 

A mechanical reduction in resolution reduces resolved porosity, even though this effect is more pronounced for carbonates [20,26,32]. This decrease was also seen here and even though while porosity decreased, permeability increased. This effect is rather contradictory, because resolution reduction should normally sever connections by smaller pores [26,29,30,31,33,36], but the pore network models actually join smaller pores together as larger pores, resulting in larger pore and throat radii and volumes [26,35]. This, in turn, results in higher permeability, as permeability is largely dependent on throat radii. Additionally, the microporous network did not appear to be restrictive to flow in this sample and increasing permeability with decreasing resolution was also mentioned by Zhang et al. [27], Keehm and Mukerji [34], and Ortega Ramírez et al. [35]. The artificially reduced resolution did not change porosity and permeability was only slightly impacted. This is because connectivity is much better retained during ARR than for MRR, as can be seen from Figure 6, the much higher coordination number in ARR (Figure 6f), and a disproportionate amount of pore throats for the amount of remaining nodes. This higher coordination number and throat-to-pore ratio for ARR is caused by the retention of thin, one-pixel width features at pore boundaries which are misclassified as pores and throats by the pore network extraction algorithm. This also causes the more accurately estimated specific surface area for ARR, although these one-pixel pores and pore throats are unrealistic artefacts from ARR. MRR has decreasing SSA because surface rugosity is lost with decreasing resolution.

During moisture absorption, higher resolution samples show faster uptake of moisture in the pore network because of the presence of smaller pores because Equation (1) shows that liquid will faster condense in smaller pores. During drying, the moisture profile (Figure 9a,b) is smoother at a higher resolution which suggests that overall smaller pores might be present, and a certain part of the larger pores is better shielded from the drying front by smaller pores which longer retain water (Equation (2)). This is the ink-bottle effect [143]. Moisture permeability follows the moisture uptake profile (Figure 9c,d) and permeability increases faster for higher resolution images during moisture absorption or decreases slower during drying. This effect is caused by the larger fraction of small pores (Figure 6a) which retain water but is also caused by the water absorbed on pore margins, which is better absorbed when pore margins are complex and contain crevices. These smaller pores and pore boundaries are better resolved at higher resolutions, as seen from the shape factor (Figure 6b,e) which is lower at better resolutions and the SSA which increases, both suggesting more complex pore boundaries. This allows fine pores and surface water to build and maintain a liquid backbone along which liquid water starts and keeps flowing. This liquid water has a much higher impact on total permeability than vapour. This absorbed water can be important in describing building materials as more water equals more corrosion, a higher risk for frost damage, and it forms a substrate for microbial build-up [17,19]. None of these things are desirable in a building stone, therefore the best possible description is required, which is obtained at higher resolutions [19]. Additionally, simulations to characterize moisture retention for groundwater analyses also benefit from using the most optimal resolution [29,36].

The evolution of relative permeability during two-phase flow is not heavily influenced by resolution (Figure 11), however, the amount of residual oil after oil drainage differs distinctly. This was also mentioned by Shah et al. [32]. Logically, whenever an oil reservoir is exploited, oil companies are more interested in how much oil can be extracted, thus this residual oil is an important characteristic. Studying a sample at a lower resolution (MRR), might result in a 44% more trapped oil: 36% of oil is trapped when scanning a sample at 12 µm, while instead 25% is trapped when studying exactly the same volume at 4 µm (Figure 12). This residual oil fraction is impacted by surface flow, in oil-wet pores, which is longer sustained when more pore boundary irregularities and crevices are present. These complexities are better resolved at higher resolution. Additionally, because some finely porous connections that possibly remained oil-wet were removed, snap-off events are more successful at trapping oil. Even though these observations cannot be directly translated to a two-phase brine-CO_2_ system, which is studied for the capillary trapping of CO_2_ [4,149], it can be seen that such major differences in trapping are important when analysing a reservoir for CO_2_ storage. Therefore, the impact of resolution is not negligible and the best possible resolution should be used for two-phase flow simulations [29,32,36]. 

The influence of ARR is much less pronounced than MRR. This is caused by the retention of pore complexities, such as small pore throats of one voxel across or relates to surface area, as seen from the decreasing shape factors (Figure 6b,e) and specific surface area. These small pores and pore complexities are responsible for better reproduction of the remaining oil fraction and the moisture uptake pattern, because small pores and surface complexities sustain oil flow over a longer water saturation range. Thus, ARR with simple voxel binning is incapable of reproducing realistic information loss that is observed with MRR. The same ARR technique was used by Keehm and Mukerji [34], Alyafei et al. [26], and Ortega Ramírez et al. [35] amongst others, but more complex methods, like that of Wang et al. [85], could mitigate this problem. However, to train neural networks it might be more desirable to work with different CT scans of one sample at different resolutions (MRR), because deployment to real situations better benefits from a well-trained network than from one trained on an easier problem. Therefore, this research prefers to train and validate the super-resolution GAN on two scans obtained at two different resolutions.

Additionally, this resolution study, even though it was only limited to one sample, has merit in research, as often, the impact of resolution is only studied, either for artificial resolution reduction [26,34,150] or for mechanically lower resolution but on different samples [20,30,151,152]. Only rarely was the effect of resolution studied on the same volume, but results and conclusions are consistent with other studies.

### 4.2. Super Resolution Models and Their Impact on Fluid Flow Simulation

Low resolution (LR) images were fed to a generative adversarial neural network to obtain images at a higher resolution thus artificially improving the resolution, also referred to as super-resolution models (SRM). Modelled results were compared to actual high resolution (HR) scans that match the LR input data, thus representing the “ground truth”. Visual simulation results show that smaller pores which are close to the resolution limit of the LR volume, are better resolved and high frequency noise that is visible in CT scans was not reproduced in SRM (Figure 4). Additionally, pore boundaries are better resolved, which is confirmed by better resolved specific surface area. Furthermore, all pore network properties, especially pore and throat count and pore and throat radius distributions are much closer to HR properties (Figure 7), although shape factor distributions (Figure 7b,e) still resemble the distribution of LR volumes and pores with lower shape factors are insufficiently reproduced. Total porosity is slightly overestimated in each volume, albeit only by a small fraction. This overestimation lies within the range of porosity distributions noted by Wang et al. [84] and is slightly higher than in Shams et al. [88].

Despite overestimation of total porosity, SRM better estimate all fluid flow properties than LR volumes do. Single-phase absolute permeability is better estimated by SRM than LR, although an overestimation between 39% and 91% remains, whereas this originally was 72%–171% for LR volumes. This better permeability estimation can be explained by the better representation of the smaller pore throats and LBM simulations show that additional connections and improved surface area contribute to more accurate predictions. The better predictions of SSA are reflected by fluid flow paths that are slowed down at pore boundaries by additional collisions on pore surface rugosity (Figure 8 h). Apart from this, additional connections that open up part of the pore network that was disconnected in LR better distributes fluid velocities (Figure 8h). This is seen from lower fluid flow velocities in larger pores of SRM (Figure 8b,e,h) than they are in LR (Figure 8a,d,g), thus closer representing HR (Figure 8c,f,i). However, the SRM still exaggerates fluid flow velocities compared to HR volumes. This overestimation is caused by slightly overestimated connectivity (Figure 8h,i) and pore surface rugosity that is underestimated (Figure 8), thus insufficiently slowing fluids down. 

The onset of moisture absorption was better predicted in SRM than for LR and started at lower log(P_c_) values (Figure 10). This lower P_c_ value suggests that smaller pores and pore throats are better represented in SRM. Despite the overestimated saturated moisture content, which is directly related to total porosity, moisture permeability evolution is better estimated for SRM and is closer to HR. This is related to the development of the liquid backbone through which fluids migrate, which is built at a lower capillary pressure, as it is in HR. Hence, the variation of moisture permeability with increasing (or decreasing) capillary pressure much more resembles the HR behaviour than the LR path (Figure 10). This early development of liquid backbone is dominated by the finer pores in the simulated volume, rather than pore boundary irregularities along which a water film could have formed. These pore surface irregularities are probably less significant because, despite a clear increase in specific surface area, shape factors of pores and pore throats are not much better represented in pore network models of SRM than they were in LR. Absorption-drying hysteresis is broader for SRM than it is for LR, much alike HR. This probably relates to a pore and throat size distribution that generally favours smaller pores, as opposed to LR (Figure 7a,g). These smaller pores improve the ink-bottle effect, thus delaying drying of larger pores [19,153]. As previously mentioned, the prediction of the absorption and drying aspects of a material are important to assess whether a material might be suitable for usage as construction material. These properties are best computed on digital material samples, as not one laboratory techniques covers the entire relevant capillary pressure spectrum [153,154,155] and analyses are often not reproducible between laboratories [12]. Therefore, such unsaturated models could benefit from the developed super-resolution workflow, although additional research is required to also represent the smallest pores because nanoporosity is relevant for construction material studies as well. 

The evolution of relative permeabilities in two-phase flow (Figure 13) did not show significant variation with resolution and neither does it when comparing SRM, LR and HR volumes. If any similarities between the volumes ought to be noted, the SRM K_r,w_ pattern during primary water drainage most resembles the pattern observed in LR. However, the preceding discussion on resolution influence showed that major differences are noted in the remaining oil fraction, as was also noted by Shah et al. [32]. This remaining oil fraction is much better represented with a 4%–40% mismatch with HR, resulting in an average (n = 4) 33.73±1.23% remaining oil for SRM, while it would be 29.38±4.65% for HR, and 43.23±1.78% for LR (Table 7). This better estimated remaining oil fraction is explained by an oil-wetting backbone that sustains flow for a longer pressure range and maintains connections for pores which might otherwise be closed off by snap-off. This backbone remains because of better resolved smaller pores and possibly better resolved pore surfaces. However, this latter property was not well represented in the shape factors of SRM thus is probably less important than the smaller pores and better estimated pore and throat radii. Nevertheless, the higher SSA might be implied in smaller pores in the network, which could be segmented as if they are separate pores and pore throats, connected to a larger pore. This incorrect segmentation into pores and pore throats should, however, not be too pronounced in the used algorithm of Raeini et al. [55]. 

Generally, the SRM pore network approximates the HR pore network, although porosity is overestimated. Despite this overestimation, the SRM pore network is much more efficient at predicting fluid flow than LR is and closely represents HR. This shows that the proposed super-resolution method based on GANs could be interesting in post-processing CT scans in order to get more adequate fluid flow estimations of larger volumes. Moreover, once the GANs are well-trained, simulations are fast and can be performed on normal computers. However, it is expected that with an increasing gap in resolution between low- and high-resolution scans, the models could further overestimate porosity in some areas, as the “uncertain voxels”, i.e., those with grayscale values between porosity and matrix are more common. The neural network, which ends with a sigmoid activation layer attempts to make a decision between matrix and porosity allowing little room for uncertainty, therefore causing the small porosity overestimation. Additionally, more fine pores could be lost when the resolution gap increases, because at lower resolutions, smaller pores would be less visible, thus further challenging the neural network to learn how to super-resolve these fine pores.

The proposed super-resolution method fits within the group of techniques that attempts to mitigate or solve the resolution versus sample-size trade-off. Up to now, these super-resolution methods have not often received attention for geological applications and were mainly addressed by Wang et al. [82,83,85] and Wu et al. [75] who attempted classical super-resolution methods like neighbour embedding [75,82,156,157,158] and sparse representation [83,159], although Wang et al. [84,85] were also the first to use convolutional neural networks to address the super-resolution problem for geological materials. Compared to these networks, the presented research used deeper neural networks, an encoder-decoder architecture with residual connections and generative training to better conform to approaches in recent super-resolution literature [93,96,100]. Additionally, Shams et al. [88] were the first to use a GAN to solve the super-resolution problem and worked directly on segmented images, of which the low resolution images were artificially prepared. Furthermore, other super-resolution studies checked the adequacy of their models with structural parameters like peak signal to noise ratio and structural similarity. However, the goal of super-resolved models is to improve fluid flow predictions and therefore this research verified that the developed SR models had improved fluid flow predictions over LR significantly and they did so for unsaturated flow and one or two-phase saturated flow.

### 4.3. Limitations and Future Perspectives

There are some limitations to this work that should be addressed in future research. We address three of them. Further, general drawbacks on the use of neural networks are addressed too. Firstly, this work provides a proof-of-concept study, which is why only one sample was tested and both training and validation data are derived from the same sample although at different locations within the sample. In future steps, this should be expanded to be able to use this workflow on unseen samples by creating a CT library. In order to do this, the challenge of grayscale variability between scans and machine related noise should be overcome. Even though the neural network architecture was optimized for this, by using instance normalization instead of batch normalization, data augmentation by grayscale and contrast variation during training could generalize the trained networks. However, the severely increased amount of data forces the usage of supercomputers with AI dedicated GPUs, whereas training on one sample was possible on a standard workstation. Once the network is trained, it could again be used on a standard computer. Secondly, the step from 12 µm to 4 µm is small albeit significant. Further steps should include the addition of higher resolution methods, like FIB-SEM. However, it should be ensured that sufficient overlap between scales remains. Thirdly, this research presents quite a simple GAN which requires supervised learning, thus needs matched datasets. The usage of cycle-GANs would allow for unsupervised learning and is promising for super-resolution applications for CT [107]. However, these cycle-GANs would require a sufficiently large GPU to train, as two GANs are trained simultaneously [107]. Furthermore, hierarchical GANs are promising in training efficiency and if a strategic sampling workflow is applied, new training data could be introduced during each increment of these hierarchical GANs [109,160].

Even though neural networks, and more particularly, generative adversarial neural networks (GAN), prove to be promising, some challenges and drawbacks were identified. Firstly, neural networks present a black-box approach, which makes it harder to identify why certain things can go wrong, like the overestimation of porosity in this research. Secondly, related to this black-box, GAN training can be unstable and sometimes it is hard to find a solution for this. Even though Radford et al. [130] provide very good suggestions to overcome training instability, building the networks of this research showed that a lower learning rate for the discriminator (10^−5^ instead of 10^−4^) was essential to overcome the problem that the low and high resolution images show similar structures. This similarity issue also required the use of a network that is not too deep and that contains short-range residual skips in order to avoid gradient loss. Additionally, batches that are fed to the discriminator could be shuffled to introduce additional randomization. Despite these suggestions, the models remain a “best guess” and imperfections, like the overestimation of porosity indicate that improvements are still needed. Most likely, this could be improved by introducing larger training datasets and image augmentation, although training such networks could not be done on a user-grade desktop, like used in this research.

## 5. Conclusions

In this paper, a super-resolution method based on generative adversarial networks is used to improve the resolution of CT scans with the goal to obtain better fluid flow simulations. First a small study verified the impact of resolution on the pore network and on the studied fluid flow properties. In general, a lower resolution results in a decreased count of pores and pore throats, while also increasing the average pore and throat sizes. Absolute single-phase permeability is overestimated severely with worsening resolution, despite constant porosity. This is a result of a lower specific surface area (SSA) and generally larger pores and pore throats. The effect of resolution on unsaturated permeability is mainly distinct in the lower capillary pressure range where moisture content and permeability start to increase at lower capillary pressures when higher resolution volumes are used. This is the result of a liquid backbone originating in the finest pores and on pore surfaces, where liquids are best captured within surface irregularities. This effect of such liquid backbone is also produced in two-phase simulations, where a wetting layer will remain in fine pores and pore surface irregularities, allowing more oil extraction during waterflooding thus resulting in decreased remaining oil fraction with improving resolution. In general, the effect of resolution is less marked in volumes with artificially reduced resolution. Therefore, in this research, neural networks are trained using samples effectively scanned at various resolutions.

The applied super-resolution method succeeds in generating visually more appealing results. Furthermore, the simulated volumes have a more realistic pore network with more pores and pore throats, compared to the low-resolution input. Additionally, smaller pores and pore throats are better characterized, although shape factors of the modelled pores are not yet approximating those of the high-resolution input, despite a clear increase in specific surface area. Single-phase fluid flow simulations show that the models present a significant improvement over low resolution input volumes. Visualization of such fluid flow shows that better connectivity, albeit overestimated, more realistically distributes fluid flow velocities and the higher surface area slows down fluids at pore boundaries. Moreover, unsaturated flow is better represented, especially in the lower capillary pressure range, where the onset of moisture uptake and the increase of moisture permeability is better predicted in super-resolved models in comparison to low resolution input. Moreover, saturated moisture permeability is better predicted despite overestimated saturated moisture content. Ultimately, two-phase flow simulations show that even though the relative permeability evolution with water fraction has not clearly changed, the remaining oil fraction is much better predicted in super-resolved models than it was in low resolution input. The improved fluid flow characteristics of all fluid transport, whether it is saturated or unsaturated single-phase flow or two-phase fluid flow are related to a better representation of the pore network and better representation of pore and throat boundary irregularities.

The applied super-resolution method could therefore be interesting as a pre-processing step in a fluid flow study, where a GAN is adequately trained to capture multiple cases. This procedure would allow researchers to take scans at lower resolutions, allowing to use larger samples or save a lot of scanning time, and then artificially increase the resolution. After a GAN is trained, potentially on a GPU-dedicated computing node of a supercomputer, it does not require heavy computing capacities to apply the super-resolution procedure on samples. However, caution is required when training a GAN, and therefore the following suggestions were made: (1) use a lower learning rate for discriminator, (2) avoid gradient loss by not making networks too deep, (3) add short-range residual skips, and (4) try to shuffle discriminator batches to increase randomization and thus stabilize training.

## Figures and Tables

**Table 1:** Training input parameters for generator and discriminator.

Parameter	Generator	Discriminator
Loss function	Mean absolute error (*mae*) and adversarial loss (100:1 ratio)	Mean squared error (*mse*)
Optimizer	Adam	Adam
Learning rate	10^−^^4^	10^−^^5^
β_1_	0.5	0.5
Batch size	4	
Epochs	500	

**Table 2:** Influence of resolution on mean pore network properties, studied for two volumes and for mechanical and artificial resolution reduction. Relative difference with expected highest resolution data is added to check the decay of properties.

Parameter	Res.	VALUES				RELATIVE TO 4 µm			
		Mechanical		Artificial		Mechanical		Artificial	
		V1	V2	V1	V2	V1	V2	V1	V2
*Amount of pores/throats*	4 µm	21088/33756	27299/43396	21089/33756	27299/43396	1.00/1.00	1.00/1.00	1.00/1.00	1.00/1.00
	8 µm	5798/9719	7406/12158	6097/13496	7724/16953	0.27/0.29	0.27/0.28	0.29/0.40	0.28/0.39
	12 µm	2762/4538	3423/5505	2801/7029	3439/8665	0.13/0.13	0.13/0.13	0.13/0.21	0.13/0.20
	16 µm	1527/2349	1920/2945	1445/3954	1732/4771	0.07/0.07	0.07/0.07	0.07/0.12	0.06/0.11
*Throats per node*	4 µm	1.60	1.59	1.60	1.59	1.00	1.00	1.00	1.00
	8 µm	1.68	1.64	2.21	2.19	1.05	1.03	1.38	1.38
	12 µm	1.64	1.61	2.51	2.52	1.03	1.01	1.57	1.59
	16 µm	1.54	1.53	2.74	2.75	0.96	0.96	1.71	1.73
*Mean Pore Volume* log(m³)	4 µm	−13.23	−13.25	−13.23	−13.25	1.00	1.00	1.00	1.00
	8 µm	−12.43	−12.45	−12.39	−12.42	0.94	0.94	0.94	0.94
	12 µm	−12.01	−12.03	−11.95	−11.96	0.91	0.91	0.90	0.90
	16 µm	−11.73	−11.75	−11.61	−11.63	0.89	0.89	0.88	0.88
*Mean Pore Shape Factor*	4 µm	0.044	0.045	0.044	0.045	1.00	1.00	1.00	1.00
	8 µm	0.048	0.049	0.040	0.040	1.08	1.09	0.90	0.90
	12 µm	0.049	0.051	0.039	0.040	1.11	1.13	0.89	0.89
	16 µm	0.051	0.052	0.038	0.039	1.15	1.16	0.87	0.88
*Mean Coordination number*	4 µm	3.14	3.13	3.14	3.13	1.00	1.00	1.00	1.00
	8 µm	3.25	3.21	4.32	4.31	1.04	1.02	1.38	1.38
	12 µm	3.16	3.12	4.88	4.94	1.00	1.00	1.55	1.58
	16 µm	2.92	2.96	5.29	5.38	0.93	0.94	1.69	1.72
*Mean Throat Radius* log(m)	4 µm	−5.04	−5.06	−5.04	−5.06	1.00	1.00	1.00	1.00
	8 µm	−4.78	−4.80	−4.89	−4.90	0.95	0.95	0.97	0.97
	12 µm	−4.64	−4.67	−4.77	−4.79	0.92	0.92	0.95	0.95
	16 µm	−4.54	−4.56	−4.67	−4.69	0.90	0.90	0.93	0.93
*Mean Throat Total Length* log(m)	4 µm	−4.32	−4.34	−4.32	−4.34	1.00	1.00	1.00	1.00
	8 µm	−4.12	−4.13	−4.12	−4.13	0.95	0.95	0.95	0.95
	12 µm	−4.02	−4.03	−4.00	−4.01	0.93	0.93	0.93	0.92
	16 µm	−3.94	−3.95	−3.89	−3.90	0.91	0.91	0.90	0.90

**Table 3:** Impact of super-resolution models on mean pore network properties, studied for four volumes of 500³ voxels each (3 first columns). Relative difference with expected high-resolution data (last 2 columns).

Parameter	Vol.	VALUES			RELATIVE TO HR	
		SRM	HR	LR	SRM	LR
*Amount of pores/throats*	V1	1956/3425	2211/3809	850/1364	0.88/0.90	0.38/0.36
	V2	2949/4896	3094/5077	1243/1708	0.95/0.96	0.40/0.34
	V3	2959/4418	3249/5014	1264/1466	0.91/0.88	0.39/0.29
	V4	2702/3818	2815/4238	1160/1476	0.96/0.90	0.41/0.35
*Throats per node*	V1	1.75	1.72	1.60	1.02	0.93
	V2	1.66	1.64	1.37	1.01	0.84
	V3	1.49	1.54	1.16	0.97	0.75
	V4	1.41	1.51	1.27	0.94	0.85
*Mean Pore Volume* log(m³)	V1	−13.13	−13.23	−12.62	0.99	0.95
	V2	−13.19	−13.24	−12.68	1.00	0.96
	V3	−13.21	−13.26	−12.75	1.00	0.96
	V4	−13.17	−13.20	−12.67	1.00	0.96
*Mean Pore Shape Factor*	V1	0.046	0.043	0.046	1.08	1.08
	V2	0.048	0.044	0.048	1.08	1.09
	V3	0.049	0.045	0.049	1.09	1.10
	V4	0.047	0.043	0.049	1.09	1.13
*Mean Coordination number*	V1	3.39	3.34	3.06	1.01	0.91
	V2	3.21	3.18	2.61	1.01	0.82
	V3	2.89	3.00	2.18	0.96	0.73
	V4	2.73	2.91	2.41	0.94	0.83
*Mean Throat Radius* log(m)	V1	−4.93	−5.02	−4.68	0.98	0.93
	V2	−4.98	−5.04	−4.71	0.99	0.93
	V3	−4.99	−5.06	−4.75	0.99	0.94
	V4	−4.97	−5.03	−4.74	0.99	0.94
*Mean Throat Total Length* log(m)	V1	−4.24	−4.30	−4.18	0.99	0.97
	V2	−4.31	−4.35	−4.23	0.99	0.97
	V3	−4.32	−4.37	−4.26	0.99	0.98
	V4	−4.32	−4.36	−4.25	0.99	0.97

**Table 4:** Impact of resolution on porosity, specific surface area and absolute permeability determined from pore network models, studied for two volumes of 1000³ voxels each. Relative difference with expected highest resolution data is added.

	Type	Vol.	Porosity				Specific Surface Area (mm²/mm³)				PNM Permeability (Darcy)			
**Resolution**			4 µm	8 µm	12 µm	16 µm	4 µm	8 µm	12 µm	16 µm	4 µm	8 µm	12 µm	16 µm
**VALUES**	Mech.	V1	0.26	0.26	0.25	0.23	15.69	10.67	8.46	6.81	13.80	22.02	29.00	35.40
		V2	0.25	0.25	0.23	0.22	18.02	12.10	9.38	7.45	3.83	6.67	8.47	10.49
	Artif.	V1	0.26	0.26	0.26	0.26	15.69	14.73	13.61	12.64	13.80	14.52	16.10	18.39
		V2	0.25	0.25	0.25	0.25	18.02	16.87	15.53	14.37	3.83	4.89	6.69	8.21
**RELATIVE TO 4µm**	Mech.	V1	1.00	1.00	0.95	0.91	1.00	0.68	0.54	0.43	1.00	1.60	2.10	2.56
		V2	1.00	0.99	0.92	0.86	1.00	0.67	0.52	0.41	1.00	1.74	2.21	2.73
	Artif.	V1	1.00	1.00	1.00	1.00	1.00	0.94	0.87	0.81	1.00	1.05	1.17	1.33
		V2	1.00	1.00	1.00	1.00	1.00	0.94	0.86	0.80	1.00	1.28	1.74	2.14

**Table 5:** Impact of super-resolution models on porosity, specific surface area and absolute permeability determined from pore network models (PNM) and from Lattice Boltzmann Method (LBM), studied for four volumes of 500³ voxels each. Relative difference with expected high-resolution data is added.

	Type	*Porosity*				*Specific Surface Area (mm²/mm³)*				*PNM Permeability (Darcy)*				*LBM Permeability (D)*
**Subvolume**		V1	V2	V3	V4	V1	V2	V3	V4	V1	V2	V3	V4	V1
**VALUES**	SRM	0.32	0.32	0.30	0.23	112.4	131.2	121.7	111.5	17.40	29.59	50.88	2.29	19.08
	HR	0.28	0.28	0.27	0.21	123.9	142.8	136.2	124.7	12.52	16.99	31.17	1.20	12.21
	LR	0.30	0.29	0.28	0.21	76.3	85.3	79.1	73.1	24.77	46.02	53.67	2.68	27.61
**RELATIVE TO HR**	HRM	1.13	1.13	1.10	1.09	0.91	0.92	0.89	0.89	1.39	1.74	1.63	1.91	1.56
	LR	1.05	1.03	1.01	1.00	0.62	0.60	0.58	0.59	1.98	2.71	1.72	2.24	2.26

**Table 6:** Impact of super-resolution models on moisture saturation, saturated moisture permeability and vapour permeability studied for four volumes of 500³ voxels each. Relative difference with expected high-resolution data is added.

Parameter	Subvolume	Values			Relative to HR	
		SRM	HR	LR	SRM	LR
*Moisture saturation*	V1	305.09	269.83	284.05	1.13	1.05
	V2	311.64	274.53	275.58	1.14	1.00
	V3	290.30	264.79	261.68	1.10	0.99
	V4	215.36	198.79	194.40	1.08	0.98
*Saturated moisture permeability*	V1	1.50 × 10^−5^	8.52 × 10^−6^	2.30 × 10^−5^	1.76	2.70
	V2	2.73 × 10^−5^	1.60 × 10^−5^	3.95 × 10^−5^	1.71	2.48
	V3	2.06 × 10^−5^	1.00 × 10^−5^	2.76 × 10^−5^	2.06	2.75
	V4	2.15 × 10^−6^	1.18 × 10^−6^	2.58 × 10^−6^	1.82	2.18
*Plateau vapour permeability*	V1	2.41 × 10^−16^	1.84 × 10^−16^	2.17 × 10^−16^	1.31	1.18
	V2	3.27 × 10^−16^	2.39 × 10^−16^	2.72 × 10^−16^	1.37	1.14
	V3	2.39 × 10^−16^	1.84 × 10^−16^	2.14 × 10^−16^	1.30	1.16
	V4	1.02 × 10^−16^	8.03 × 10^−17^	7.41 × 10^−17^	1.27	0.92

**Table 7:** Impact of super-resolution models on residual oil fraction and relative water permeability after waterflooding from two-phase oil-water fluid flow simulations on pore network models on four volumes of 500³ voxels each. Relative difference with expected high-resolution data is added. In the fourth volume, SRM relative water permeability appears severely overestimated.

	Type	*Relative Water Permeability (after Water-Flooding)*				*Residual Oil Fraction*			
**Subvolume**		V1	V2	V3	V4	V1	V2	V3	V4
**VALUES**	SRM	0.21	0.71	0.69	6.0 × 10^−3^	0.32	0.33	0.35	0.35
	HR	0.05	0.76	0.83	2.2 × 10^−7^	0.28	0.29	0.25	0.36
	LR	3.8 × 10^−9^	0.61	0.29	5.5 × 10^−9^	0.43	0.43	0.46	0.41
**RELATIVE TO HR**	SRM	4.09	0.94	0.83	27718	1.15	1.16	1.40	0.96
	LR	7.7 × 10^−8^	0.80	0.35	0.03	1.56	1.48	1.82	1.15

## Supplementary Materials

The following are available online at https://www.mdpi.com/1996-1944/13/6/1397/s1, Figure S1: Pore network properties for influence of resolution, Figure S2: Pore network properties for super-resolution model validation, Figure S3: Influence of resolution on unsatured fluid flow, Figure S4: Super-resolution model validation for unsaturated fluid flow, Figure S5: Influence of resolution and super-resolution models on two-phase flow models.
