# Supplementary material for: Computed Tomography 3D Super-Resolution with Generative Adversarial Neural Networks: Implications on Unsaturated and Two-Phase Fluid Flow
Source: Materials (Basel). 2020 Mar 19;13(6):1397. doi: 10.3390/ma13061397 (PMC7143904; doi:10.3390/ma13061397)
Supplement: Supplementary file 1 [file materials-13-01397-s001.pdf]

materials

Supplementary Materials

Computed Tomography 3D Super-Resolution with Generative Adversarial Neural Networks: Implications on Unsaturated and Two-Phase Fluid Flow

Nick Janssens [1,*], Marijke Huysmans[1,2] and Rudy Swennen [1]

[1] Department of Earth- and Environmental Sciences, Katholieke Universiteit Leuven, Celestijnenlaan 200E, 3001 Leuven, Belgium; rudy.swennen@kuleuven.be
[2] Hydrology and Hydraulic Engineering, Vrije Universiteit Brussel, Pleinlaan 2, 1050 Brussel, Belgium; mhuysman@vub.ac.be
* Correspondence: nick.janssens@kuleuven.be

Volume 1: fake

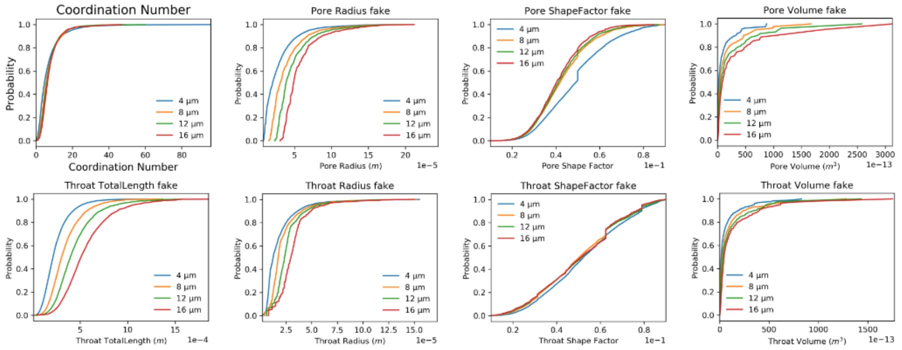

Volume 1: real

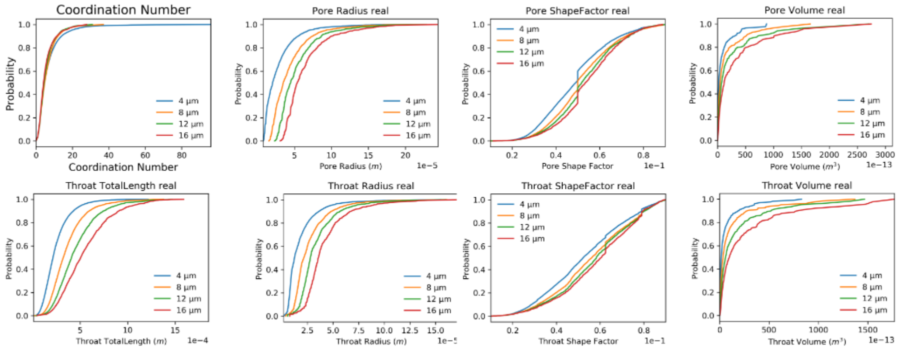

Volume 2: fake

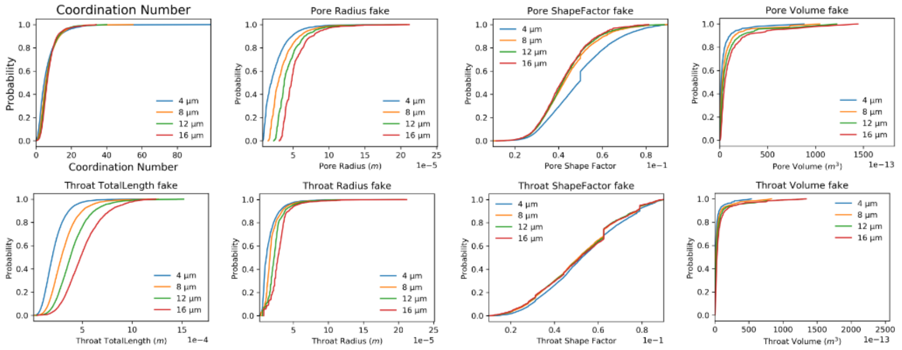

Volume 2: real

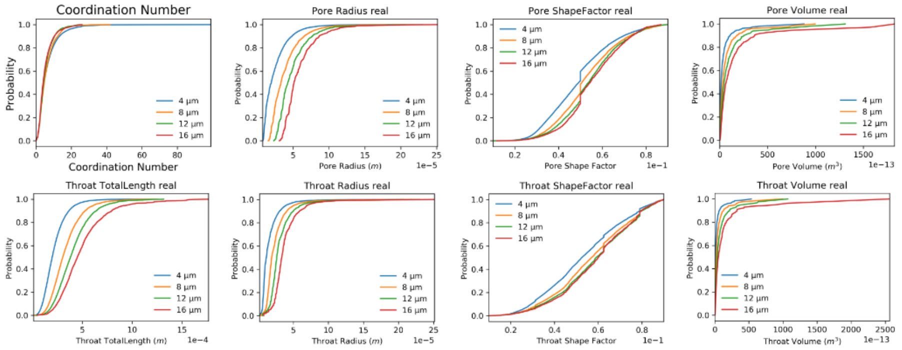

Figure S1: Pore network properties for influence of resolution.

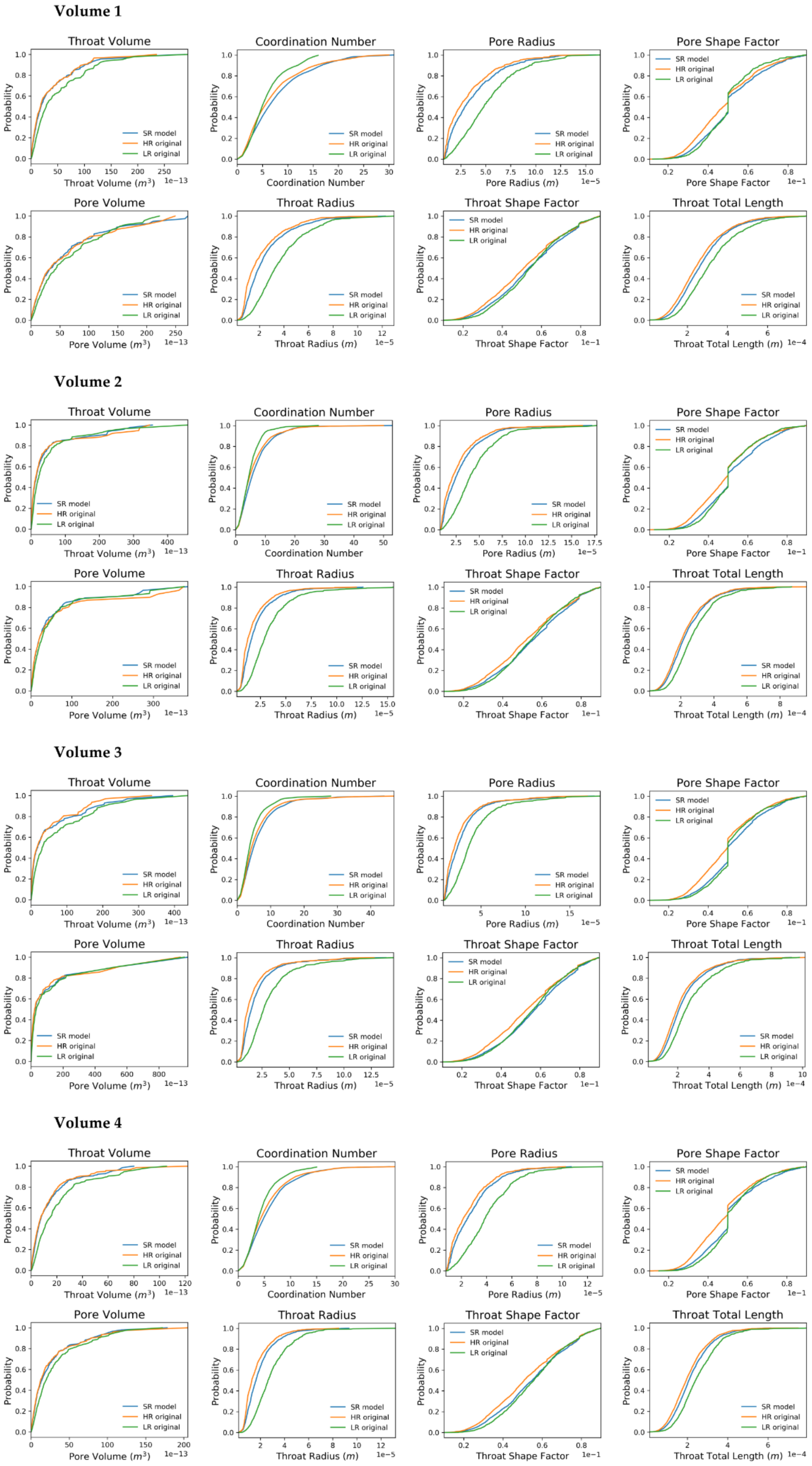

Figure S2: Pore network properties for super-resolution model validation.

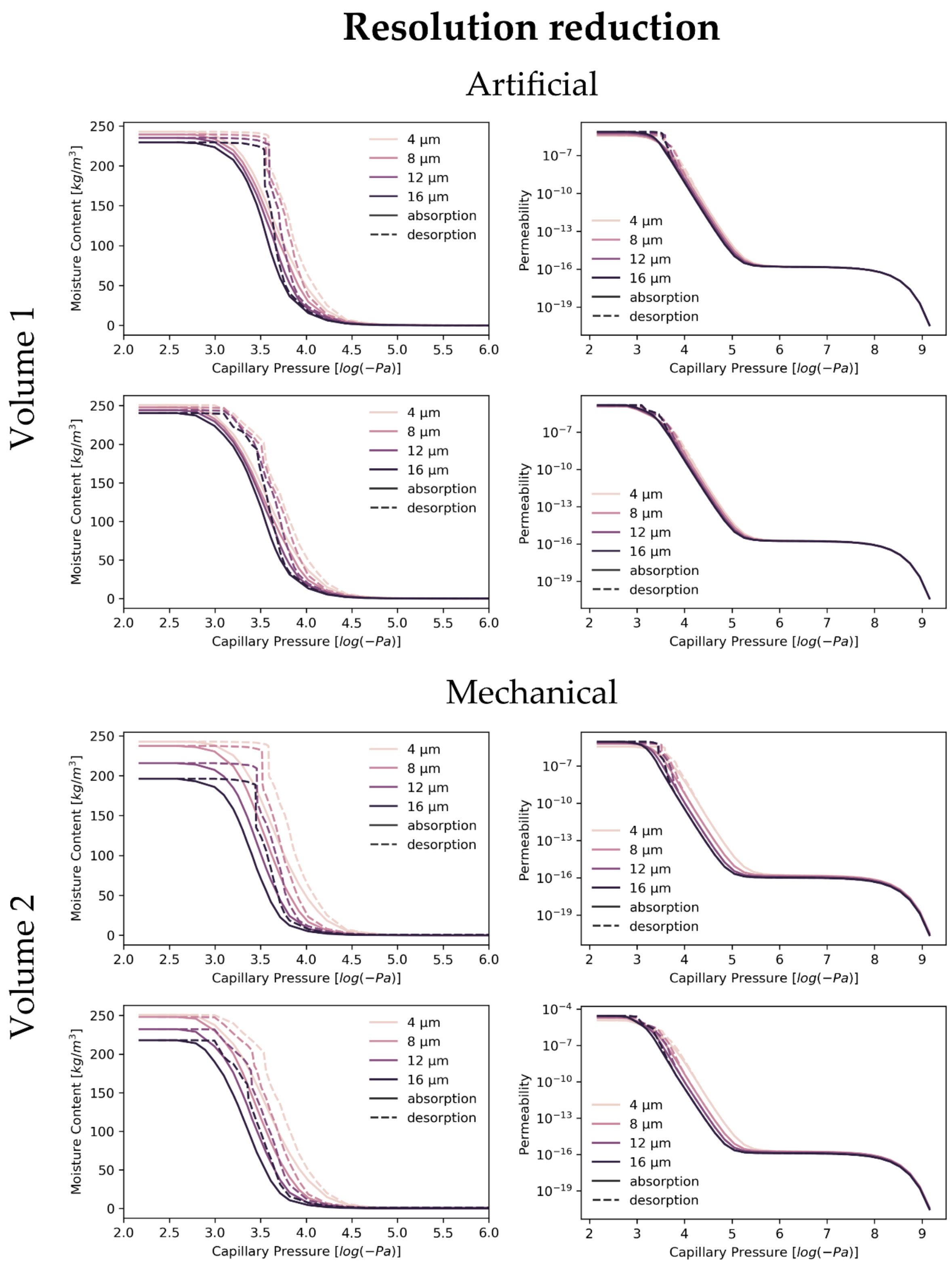

Figure S3: Influence of resolution on unsatured fluid flow.

Model validation

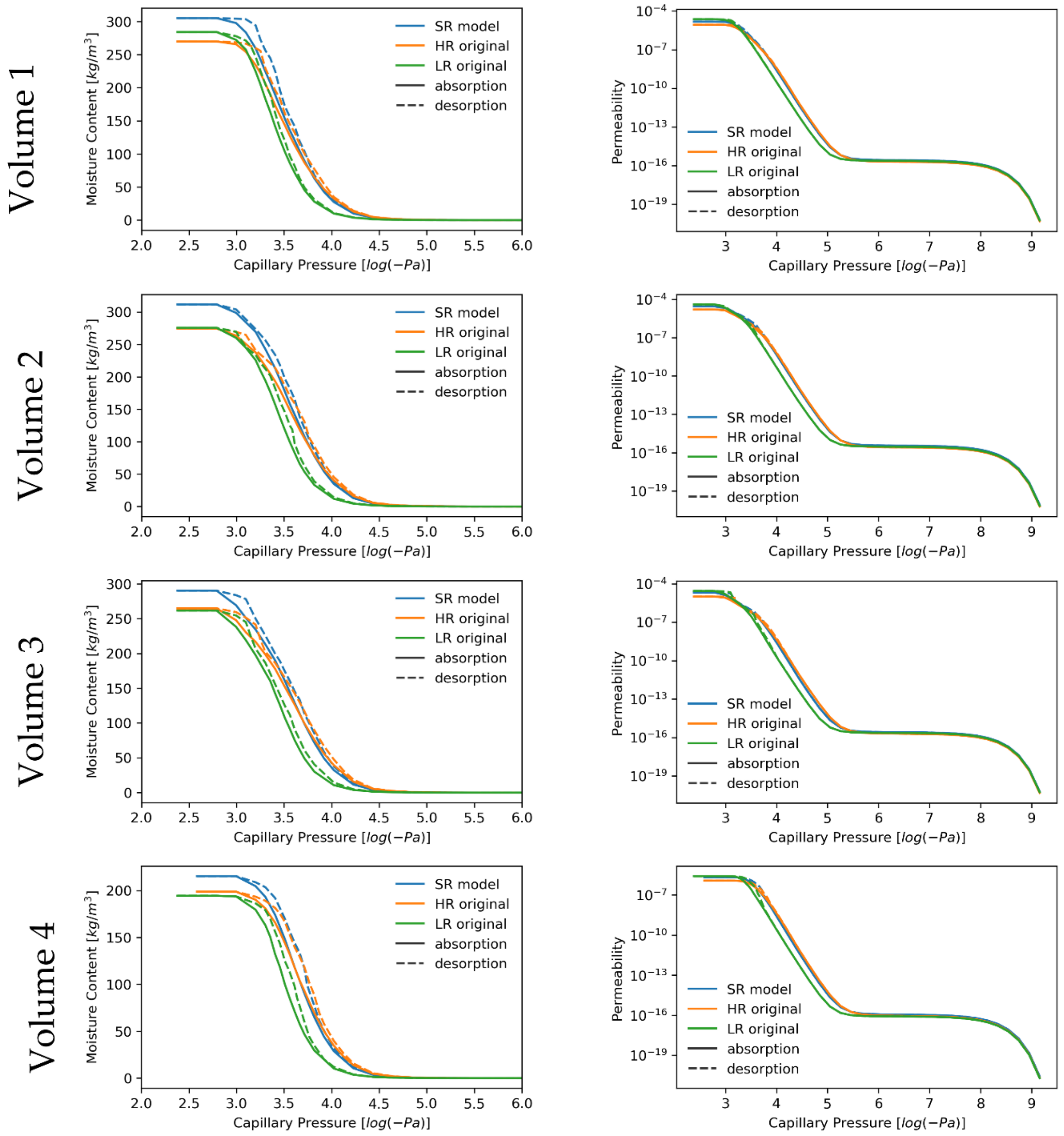

Figure S4: Super-resolution model validation for unsaturated fluid flow.

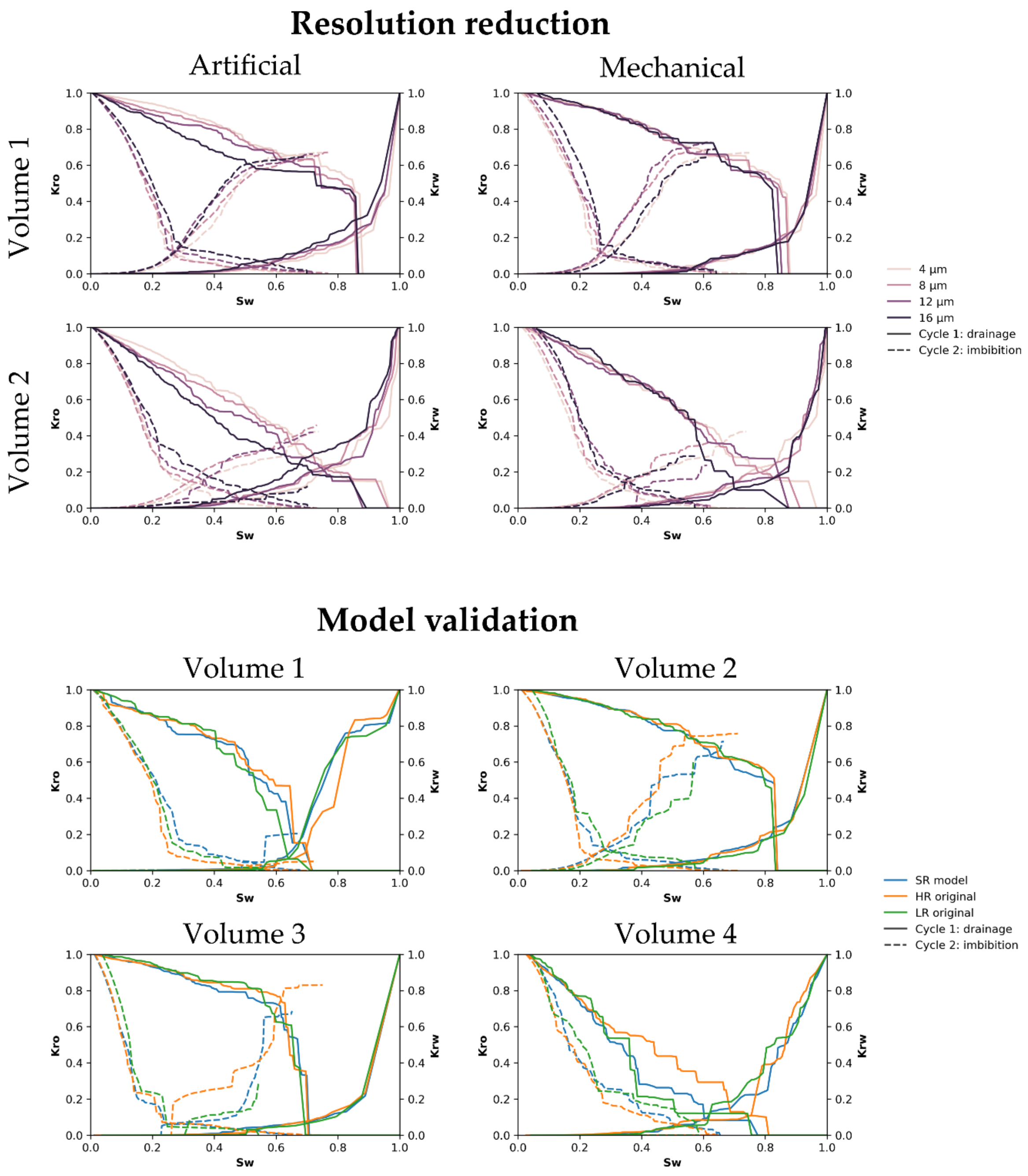

Figure S5: Influence of resolution and super-resolution models on two-phase flow models.
